# Histone acetyltransferase CSRP2BP promotes the epithelial–mesenchymal transition and metastasis of cervical cancer cells by activating N-cadherin

**DOI:** 10.1186/s13046-023-02839-2

**Published:** 2023-10-17

**Authors:** Xiaohui Yang, Fei Sun, Yueying Gao, MengYongwei Li, Mian Liu, Yunjian Wei, Qiuling Jie, Yibing Wang, Jiaoqi Mei, Jingjing Mei, Linna Ma, Yuechuan Shi, Manling Chen, Yongsheng Li, Qi Li, Mingyao Liu, Yanlin Ma

**Affiliations:** 1 Hainan Provincial Key Laboratory for Human Reproductive Medicine and Genetic Research, Haikou Key Laboratory for Preservation of Human Genetic Resource, Department of Reproductive Medicine, Key Laboratory of Reproductive Health Diseases Research and Translation, Ministry of Education, the First Affiliated Hospital of Hainan Medical University, Hainan Medical University, Haikou, Hainan 571101 China; 2 Hainan Provincial Clinical Research Center for Thalassemia, National Center for International Research, the First Affiliated Hospital of Hainan Medical University, Haikou, Hainan 571101 China; 3 Department of Obstetrics and Gynecology, Reproductive Medicine, Nanfang Hospital, Southern Medical University, Guangdong, 510515 China; 4 College of Biomedical Information and Engineering, Hainan Medical University, Haikou, 571199 China; 5 Department of Obstetrics and Gynecology, the First Affiliated Hospital of Hainan Medical University, Hainan Medical University, Haikou, Hainan 571101 China; 6 Hainan Modern Women and Children’s Hospital, Reproductive Medicine, Haikou, Hainan 571101 China; 7 Institute of Biomedical Sciences and School of Life Sciences, East China Normal University, Shanghai, China

**Keywords:** Cervical cancer, CSRP2BP, SMAD4, N-cadherin, EMT, Metastasis

## Abstract

**Background:**

Dysregulated epithelial–mesenchymal transition (EMT) is involved in cervical cancer metastasis and associated with histone acetylation. However, the underlying molecular mechanisms of histone acetylation in cervical cancer EMT and metastasis are still elusive.

**Methods:**

We systematically investigated the expression patterns of histone acetylation genes and their correlations with the EMT pathway in cervical cancer. The expression of CSRP2BP among cervical cancer tissues and cell lines was detected using Western blotting and immunohistochemistry analyses. The effects of CSRP2BP on cervical cancer cell proliferation and tumorigenicity were examined by cell growth curve, EdU assay, flow cytometry and xenotransplantation assays. Wound healing assays, transwell migration assays and pulmonary metastasis model were used to evaluate the effects of CSRP2BP on cell invasion and metastasis of cervical cancer cells in vivo and in vitro. RNA-seq, chromatin immunoprecipitation (ChIP), co-immunoprecipitation (Co-IP) and luciferase reporter assays were used to uncover the molecular mechanisms of CSRP2BP in promoting cervical cancer EMT and metastasis.

**Results:**

We prioritized a top candidate histone acetyltransferase, CSRP2BP, as a key player in cervical cancer EMT and metastasis. The expression of CSRP2BP was significantly increased in cervical cancer tissues and high CSRP2BP expression was associated with poor prognosis. Overexpression of CSRP2BP promoted cervical cancer cell proliferation and metastasis both in vitro and in vivo*,* while knockdown of CSRP2BP obtained the opposite effects. In addition, CSRP2BP promoted resistance to cisplatin chemotherapy. Mechanistically, CSRP2BP mediated histone 4 acetylation at lysine sites 5 and 12, cooperated with the transcription factor SMAD4 to bind to the SEB2 sequence in the *N-cadherin* gene promotor and upregulated N-cadherin transcription. Consequently, CSRP2BP promoted cervical cancer cell EMT and metastasis through activating N-cadherin.

**Conclusions:**

This study demonstrates that the histone acetyltransferase CSRP2BP promotes cervical cancer metastasis partially through increasing the EMT and suggests that CSRP2BP could be a prognostic marker and a potential therapeutic target for combating cervical cancer metastasis.

**Supplementary Information:**

The online version contains supplementary material available at 10.1186/s13046-023-02839-2.

## Background

Cervical cancer is the fourth most common gynecological tumour and the second leading cause of cancer-related death in women worldwide, particularly in low- and middle-income countries [1]. In 2020, an estimated 604 000 new cases of cervical cancer were diagnosed, and 342 000 deaths occurred worldwide due to this malignancy [2]. The failure of cervical cancer therapy and poor outcomes are mostly due to the development of local invasion and distant metastasis [3]. A large amount of evidence has demonstrated that epithelial-mesenchymal transition (EMT) plays a key role in the progression and metastasis of cervical cancer [4]. Dysregulation of EMT-associated genes, such as N-cadherin, E-cadherin and Twist1, induces the dysregulation of EMT and contributes to the invasion and metastasis of cervical carcinoma [4–6]. N-cadherin, as a marker of ongoing EMT, is widely recognized as a pivotal factor involved in cellular adhesion and tumour metastasis [7]. Overexpression of N-cadherin is associated with high aggressiveness and motility of tumour cells by promoting EMT, which was observed in a range of tumours such as cervical cancer, melanoma and non-small cell lung cancer [8–10]. On the other hand, histone acetylation is highlighted as one of the most important regulators of cancer EMT progression [11]. However, the mechanisms whereby specific histone acetyltransferases regulate the progression of EMT in cervical cancer are poorly understood.

Histone acetylation impacts transcriptionally active euchromatin and is regulated by the coordination between histone acetyltransferases (HATs) and histone deacetylases (HDACs) [11]. Histone acetylation has a key role in the dysregulation of EMT-associated genes and represents an essential mechanism of cancer progression and metastasis [12–14]. Wang et al.reported that Tip60 histone acetyltransferase mediates the acetylation of SPZ1 and Twist1, promoting EMT progression in liver cancer [15]. Hou et al.demonstrated that HDACs inhibit the acetylation and transcriptional activity of p65 and negatively regulate the EMT process in gastric cancer [16]. Histone acetylation also plays an essential role in the development of cervical cancer. For example, the expression levels of histone H3 acetyl K9 and histone H3 trimethyl K4 are strongly correlated with the prognosis of cervical cancer patients [17]. In addition, HAT GCN5 was reported to contribute to cell cycle proliferation in HPV-16 E7-expressing cells, indicative of its role in cervical cancer [18]. However, to what extent histone acetylation plays a role in cervical cancer metastasis by regulating the EMT process remains to be explored.

In this study, we comprehensively analysed the expression patterns of histone acetylation genes in 128 cervical tissue specimens. We discovered that seven histone acetylation genes can potentially regulate the EMT pathway, including CSRP2BP, LPCAT1, NAT14, CREBBP, MSL3, ATF2 and GNPNAT1. Among these genes, CSRP2BP (also known as lysine (K) acetyltransferase 14, KAT14) was markedly overexpressed in cervical cancer tissues and cervical cancer cell lines and significantly associated with a poor prognosis and metastasis in cervical cancer patients. Overexpression of CSRP2BP significantly promoted cervical cancer cell proliferation, migration, invasion and resistance to cisplatin chemotherapy. We further identified that HAT CSRP2BP mediated EMT signalling and cervical cancer metastasis by interacting with SMAD4 to form a complex to activate N-cadherin transcription*.* Notably, CSRP2BP overexpression increased the acetylation level of H4 at the N-cadherin promoter. Our study suggests that HAT CSRP2BP is a key regulator of cervical cancer EMT and metastasis and may be a potential therapeutic target for cervical cancer.

## Materials and methods

### Gene expression profiles of cervical cancer

Gene expression profiles of cervical cancer were obtained from Gene Expression Omnibus (GEO) under the accession number GSE63514 [19]. In total, transcriptomes of 128 frozen cervical samples spanning normalcy, cervical intraepithelial neoplasia (CINI-CINIII) lesions, and cervical cancer were analyzed in this study [19]. Gene expression profiles were measured by Affymetrix U133 Plus 2.0 microarray platform. The processed data were downloaded for further analysis.

### Collection of histone acetylation-related genes

The histone acetylation-related genes were obtained from gene ontology (GO) [20]. The genes in GO term ‘ACETYLTRANSFERASE_ACTIVITY’ were downloaded from MSigDB database [20]. In total, 88 genes were expressed in the transcriptomes of cervical cancers.

### EMT scores

To calculate the EMT score for each sample, we first obtained the EMT-related genes from MSigDB [21]. Next, we performed the single sample gene set enrichment analysis (ssGSEA) to calculate EMT enrichment score for each patient [22].

### Prioritization of genes in cervical cancer

To identify the histone acetylation-related genes that potentially correlated with EMT, we performed a two-step method to prioritize the genes. First, differential expression analysis was performed for patients with different states. Wilcoxon’s rank sum test was used to analyze the differences between the two states of patients in cervical cancer. Genes with a *p* value ≤ 0.05 was considered as differentially expressed genes in cervical cancer. For all histone acetylation-related genes, we calculated the rank score R based on the combination of fold-change and *p*-value:

$${R}_{i }=-log10\left({\mathrm{p}}_{i}\right)*log2\left({FC}_{i}\right)$$

where ${\mathrm{p}}_{i}$ is *p*-value for gene *i* and ${FC}_{i}$ is the fold-change comparison between cancer and normal samples.

Next, we identified the genes of which expressions were correlated with EMT scores in cervical cancer. Spearman correlation coefficients were calculated between the expressions of histone acetylation-related genes and EMT scores. Genes with a *p* value ≤ 0.05 and absolute spearman correlation coefficient (SCC) ≥ 0.3 were identified. Finally, genes with top ranked *R* scores and correlated with EMT scores were overlapped.

### Gene set enrichment analysis

To identify the pathways potentially correlated with gene of interest, we first calculated the SCC between the expressions of candidate gene and all other genes. All protein coding genes were ranked based on the SCC and subjected to gene set enrichment analysis (GSEA) [22]. The EMT pathway was used as the pathway for analysis.

### Tissue specimens

A total of 208 paraffin-embedded human cervical cancer tissues were obtained from the Southern Medical University Nanfang Hospital, Hainan Provincial People's Hospital and the First Affiliated Hospital of Hainan Medical University between January 2010 and December 2016. Nineteen additional matched pairs of fresh cervical cancer tissue specimens (T) and adjacent noncancerous tissue (ANT) samples were obtained from the Department of Gynecology of Hainan Provincial People's Hospital from June to July 2019. All patients involved in this study were selected from the database and histologically confirmed as having cervical cancer, and none of these patients were treated with radiotherapy, immunotherapy or chemotherapy before surgery. For total protein isolation, 19 matched pairs of fresh cervical cancer tissue and ANT samples were obtained from patients immediately after surgery and snap-frozen at − 80 °C until use. The percentages of tumour purity in these tissues used for protein analyses were established by routine histopathological analyses [23]. The study was approved by the Research Ethics Committee of the Southern Medical University Nanfang Hospital, Hainan Provincial People's Hospital and First Affiliated Hospital of Hainan Medical University IRB (HYLL-2020–060). Informed consent was obtained from each participant.

### Immunohistochemistry (IHC)

IHC was performed on paraffin-embedded human cervical tissue, ANT and tumour xenograft sections as previously described [23]. Primary antibodies, including anti-CSRP2BP (1:400; LS-B11653; LSBio), anti-N-cadherin (1:300; 13116S; Cell Signaling Technology), anti-E-cadherin (1:300; 3195; Cell Signaling Technology), and anti-Ki67 (1:400; sc-15402; Santa Cruz Biotechnology), were used to detect specific protein expression. The procedure which was performed without any primary antibody were used as a positive control. CSRP2BP staining was scored by two different pathologists who acted independently with regard to the evaluation of the intensity of staining and the proportion of positive staining. The staining index for CSRP2BP expression in cervical cancer was calculated by multiplying the two scores of the staining intensity and the proportion of positive cells. The median of all scores was used as a cut-off value for CSRP2BP. The optimal cut-off value was used as follows: a score of ≥ 6 was used to define tumours with high CSRP2BP expression, and a score of ≤ 4 indicated low CSRP2BP expression.

### Isolation and culture of primary cervical cancer cells

The surgical specimens were collected from patients with cervical cancer, cut into ~ 1 mm pieces and digested with 0.25% trypsin-250 followed by collagenase I. After then, digested tissues were pooled and washed in DMEM/F12 medium containing 10% FBS. After filtration with 200 mesh sieves, the cell suspensions were re-suspended with DMEM medium containing 10% FBS and seeded into culture flasks coated with polylysine. Medium was replenished every 24 h, and passage was performed when the cells reached 80% confluency.

### Cell culture

The human cervical cancer cell lines Hela (HPV18 +), SiHa (HPV -) and C-33A (HPV16 +) were obtained from the Chinese Academy of Sciences Cell Bank (Shanghai China), and primary human cervical cancer cells (T1, T2, T3) were established based on previously reported methods [24]. All cell lines were subjected to high glucose Dulbecco’s modified Eagle’s medium (DMEM, Gibco, CA, USA) supplemented with 10% foetal bovine serum (FBS, Gibco, CA, USA) and antibiotics (50 U/ml penicillin and 50 μg/ml streptomycin; both from Gibco, CA, USA) and maintained in 5% CO_2_ and 37 °C atmosphere.

### Plasmid construction and transfection

The coding sequence of the human CSRP2BP gene (NM_001392073, Origene, USA) was amplified and subcloned into the Xhol and BamHI sites of the pLVX-AcGFP-N1 lentiviral vector (PT3994-5, Clontech, USA) to generate the CSRP2BP expression plasmid. The Agel and EcoRI sites of the GV248-EGFP-puromycin lentiviral vector (GIDE77111, GENE, CN) were used to generate CSRP2BP shRNA constructs. The human CDH2 gene (NM_001308176, Origene, USA) was subcloned into the EcoRl and BamHl sites of the pSin-EF1α-puro lentiviral vector (SBI, USA). The coding sequence of the human H4 gene (NM_003548.2, Origene, USA) was subcloned into the EcoRl and BamHl sites of the pSin-EF1α-puro lentiviral vector (SBI, USA) to generate H4 expression plasmid. CSRP2BP mutants lacking the HAT domain (711–714 aa deleted), N-cadherin-Luc reporter mutants lacking the SEB2 and H4 mutants lacking the K5 site plasmids were generated using the KOD Plus Mutagenesis kit (Cade No. SMK-101, TOYOBO, Japan) according to the manufacturer’s instructions.

The vectors pMD2.G and psPAX2 were packaged in 293 T cells using calcium phosphate transfection. Then, transduced cells were selected for 7 days with puromycin (P7255-25MG, Sigma, USA). The surviving cells were amplified by monoclonal culture. Hela and C-33A stable cell lines expressing CSRP2BP and shCSRP2BP were established (Hela/C-33A-CSRP2BP, Hela/C-33A-Vector, Hela/C-33A-shCSRP2BP and Hela/C-33A-shcon). Protein and mRNA of transfected cells were taken for real time-PCR and Western blotting analyses. The primers used in this study are listed in supplemental Table S1.

### Western blotting

Western blotting was performed as described previously [25] by using anti-CSRP2BP (LifeSpan BioSciences, USA), anti-H4, anti-acetylated H4 on lysine 5 (H415Kac), anti-acetylated H4 on lysine 12 (H412Kac) (Abcam, USA), and anti-N-cadherin (Cell Signaling Technology, MA) antibodies. Next, the membranes were incubated with secondary antibody for 1 h at RT, and then incubated with chemiluminescent substrate kit reagents (Millipore, USA); images were captured on a Tanon 4600SF instrument (Shanghai, China). The antibodies used in this study are listed in supplemental Table S2.

### RNA isolation and quantitative real-time PCR

Total RNA was extracted using TRIzol reagent (Invitrogen Life Technologies, USA) based on standard procedures. Complementary DNA (cDNA) was prepared with the Prime Script® RT reagent kit (Takara, Japan) according to the manufacturer’s instructions. The expression level of mRNA was quantified using a SYBR® Premix E2x Taq TM II kit (Takara, Japan). The relative expression level was determined by normalizing the expression level of each target to GAPDH, and the relative mRNA fold change was determined using the 2^(−∆∆Ct)^ method. Three independent experiments were performed with each carried out in triplicate. The primers used in this study are listed in supplemental Table S3.

### Cell proliferation examination

For the cell growth curve, 1 × 10^4^ cells were seeded to 6-well plates and cultured for 6 days. The cells were counted every day to draw the cell growth curve. The 5-ethynyl-2’-deoxyuridine (EdU) assay was performed with the Cell-Light EdU Apollo 567 kit (RiboBio, China) according to the manufacturer’s instructions. All images were photographed with a fluorescence microscope (Olympus, DP72). EdU quantification was performed as the ratio of the number of red fluorescence cells, i.e., EdU + , to the total number of DAPI + cells in a given field. At least six randomly selected fields from each sample were scored. All experiments were independently performed three times, totally 18 fields to analyze. For colony formation, cells were harvested and seeded into 6-well plates with amount of 1 × 10^3^ cells/well. On day 10, the cell colonies were stained with Giemsa stain for 15 min after fixation with 4% paraformaldehyde (Biosharp, China) for 30 min. Colonies with > 50 cells were counted. Three independent experiments were performed.

### Wound healing assay

Wound healing assays were established by using Ibidi Culture Insert chambers (Ibidi, Germany) following the manufacturer’s protocol. Briefly, a total of 45 × 10^4^ cells were added to each well of the chamber. After 24 h, wound closure was monitored, and images were captured at different time points using an Olympus microscope (Olympus). Data were analyzed by using previously published methods [25].

### Transwell migration assays

Cell invasion assays were performed using 12-well tissue culture plate inserts (8.0 μm pores, BIOFIL, China) precoated with Matrigel (BD Biosciences). First, cells were suspended in 200 μL of serum-free medium and plated in the upper chambers, whereas 600 μL of medium supplemented with 10% FBS was placed in the lower chambers. After 24 h of incubation, the cells on the lower surface of the membrane filter were fixed and stained and then counted with an inverted microscope (Olympus, IX71).

### Immunofluorescence

Cells grown on cover slides were fixed with methanol and acetone (1:1) for 20 min at -20 °C, permeabilized with 0.5% Triton X-100 for 20 min, blocked with 5% BSA (Beyotime, China) in phosphate buffered saline (PBS) containing 0.1% Tween-20 for 1 h, and then incubated with a primary antibody at 4 °C overnight. Subsequently, the slides were incubated with Alexa Flour® 488 IgG anti-mouse (Abcam, US) or Alexa Flour® 594 IgG anti-rabbit (Abcam, US) at room temperature for 1 h. DAPI was used to stain the nuclei for 5 min. Fluorescence images were taken using the confocal microscopy (Olympus Fluoview FV3000).

### Flow cytometry and chemoresistance model in vitro

Flow cytometry was performed using a BD FACS Aria II cell sorter (Becton Dickinson, San Jose, CA) to analyse the cell cycle through propidium iodide (Sigma, China) staining. Briefly, cells were plated in 6-well plates (1 × 10^5^ cells/well) and cultured until they reached 90% confluency. Through flow cytometry, the annexin V + /PI cells were analysed after the indicated cells were treated with cisplatin (20 μg/ml, 40 μg/ml, 80 μg/ml) for a 24 h culture. Modfit LT 3.1 trial cell cycle analysis software or FlowJo software was used to analyse the cell cycle.

### Coimmunoprecipitation (Co-IP) assay

Plasmids expressing flag-HA-tagged SMAD3 and flag-HA-tagged SMAD4 were cotransfected into Hela cells with HA-tagged CSRP2BP by using Lipo2000™ Transfection Reagent (Invitrogen, CA, USA) according to the manufacturer’s instructions. Hela cells were lysed with RAPI (Biosharp, China) lysis buffer containing 1 × PMSF (Cell Signaling Technology, MA, USA) and 1 × protease inhibitor cocktail (Cell Signaling Technology, MA, USA). The supernatant was collected after centrifugation, and then incubated with the anti-flag antibody at 4 °C for 2 h. The antigen–antibody complex was precipitated with protein A/G (Invitrogen, CA, USA), and Western blotting was performed to examine the target proteins.

Plasmids expressing flag-HA-tagged H4 and flag-HA-tagged H4K5 mutants were respectively transfected into Hela-CSRP2BP cells using Lipo2000™ Transfection Reagent (Invitrogen, CA, USA) according to the manufacturer’s instructions. After 48 h, the supernatant was collected and incubated with the anti-flag antibody at 4 °C overnight. The antigen–antibody complex was precipitated with protein A/G (Invitrogen, CA, USA), and Western Blot was followed to examine the expression of H4Ac.

### siRNA transfection

siRNA duplexes against N-cadherin and SMAD4 were transfected into Hela-CSRP2BP, Hela-Vector cells by using Lipofectamine™ RNA iMAX Transfection Reagent (Invitrogen, CA, USA) according to the manufacturer’s instructions. The siRNA duplex sense sequences were as follows: si–N-cadherin: 5’-CCAGUGACUCUUAAGAGAA-3’, si-SMAD4: 5’-CCACCAAGUAAUCGUGCAU-3’.

### Luciferase reporter assays

The region of the CDH2 promoter (~ 2 kb) was amplified using the primers and subcloned into the PLG3-basic luciferase reporter plasmid to synthesize the pGL-3-N-cadherin promoter plasmid. The coding sequences of the human CSRP2BP gene (NM_001392073, Origene, USA) and SMAD4 gene (NM_000018.10, Origene, USA) were amplified and subcloned into the Xhol and BamHI sites of the PCGN vector (PT3994-5, Clontech, USA). Luciferase activity was measured as described previously [26].

### Xenograft models

BALB/c-nu mice (*n* = 20, 5–6 weeks of age, Gem Pharmatech. Co. LTD) were kept in aseptic conditions under constant temperature and humidity. The mice were randomly divided into four groups, and each mouse received a groin subcutaneous injection of a 100 μL suspension of 1 × 10^6^ cells. Tumour growth was detected with callipers every 2 days, and tumour volume was calculated using the following formula: Volume = (length × width^2^)/2. Magnetic resonance imaging (MRI) was used to evaluate the tumours in the mice. To further evaluate the metastatic potential of CSRP2BP, mice (*n* = 5) were transplanted with 1 × 10^6^ cells suspended in 100 μL PBS by tail intravenous injection. Six weeks after transplantation, all mice were sacrificed, and the tumours were harvested and imaged by a chemiluminescent imaging system (Sacecreation).

### Chromatin immunoprecipitation (ChIP)

Cells were fixed with 37% formaldehyde for 10 min, treated with 0.125 M glycine for 5 min and centrifuged at 3000 × g at 4 °C for 5 min to collect the crude nuclear fraction. The nuclear pellet was incubated with 1% SDS lysis buffer and sonicated to shear genomic DNA into 100 ~ 400 bp fragments. Genomic DNA fragments were transferred to slide-A-Lyzer™ G2 (Invitrogen Life Technologies, CA, USA) at 4 °C for 4 h. Immune complexes were precipitated with protein A/G beads (Invitrogen Life Technologies, USA), and soluble chromatin complexes were immunoprecipitated with human IgG antibody (Proteintech), H4ac antibody (Active Motif) or CSRP2BP antibody (Life Span Biotechnology) in ChIP dilution buffer overnight at 4 °C. The beads were sequentially washed with a low salt buffer, a high salt buffer, LiCl wash buffer, and TE buffer. The immunoprecipitated chromatin complexes were eluted in ChIP direct elution buffer at 65 °C for 30 min and incubated at 65 °C overnight to cross-link the chromatin complexes. DNAs were isolated using a QIAGEN DNA kit (28,106, Germany). The extracted DNA was analysed by real-time PCR. Primers to detect N-cadherin promoter occupancy were listed in Supplemental Table S4.

### RNA-seq

Total RNA from cells for RNA sequencing was isolated using TRIzol reagent (Invitrogen Life Technologies, USA) based on standard procedures. RNA quality was evaluated with an Agilent 2100 bioanalyzer and a NanoDrop 2000. Libraries were constructed using the standard Illumina library construction process. Each library was sequenced on an Illumina NovaSeq 6000 in 150 PE mode by Beijing Berry Genomics Co., Ltd. (Beijing, China).

### Statistical analyses

All statistical analyses were performed using the SPSS software package (version 19.0, SPSS, Inc.) and Prism 5.0 software (GraphPad, La Jolla, CA, USA). Data are presented as the mean ± standard deviation (SD) of at least three independent experiments. The independent sample *t test* was used for comparing data of groups to identify significant differences. The Kaplan‒Meier method was used for progression-free survival (PFS) and overall survival (OS) analysis, and significance was determined by the log-rank test. Multivariate logistic regression was performed to identify the independent risk factors related to the prognosis of cervical cancer. The relationships between CSRP2BP expression level and clinicopathological features were tested by the χ2 test or Fisher’s exact test. The differences were considered statistically significant at *P* < 0.05.

## Results

### Integrative analyses identify strong correlation of CSRP2BP with cervical cancer progression and EMT

**Fig. 1:**
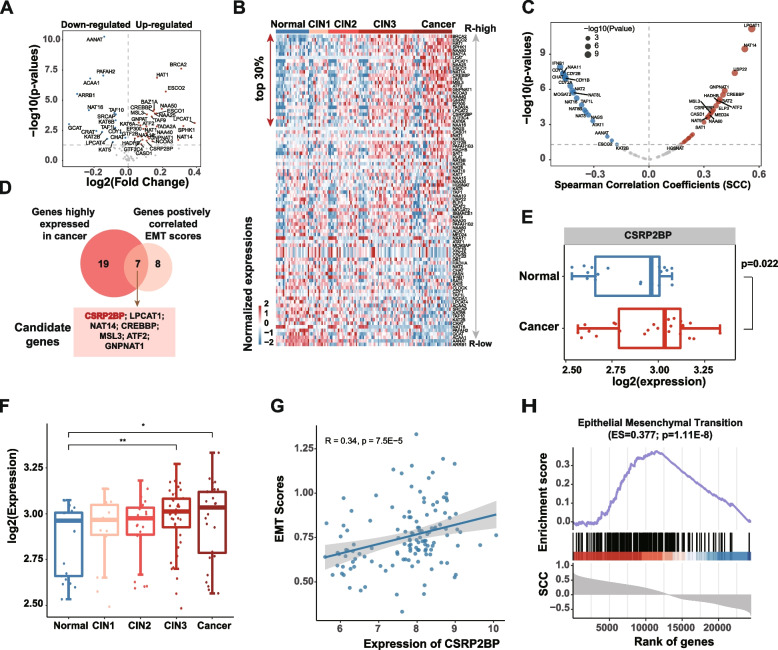
Integrative analyses identify CSRP2BP in cervical cancer. **A** Volcano plot showing the genes differentially expressed between normal and cancer patients. Blue dots for down-regulated genes and red for up-regulated genes. **B** Heat map showing the expressions of genes ranked by *R* scores. **C** Volcano plot showing the correlations between the expressions of genes and EMT scores. Blue dots for negatively correlated genes and red dots for positively correlated genes. **D** Venn plot showing the overlap between top 30% ranked genes and the genes associated EMT scores. **E** Boxplots showing the expressions of CSRP2BP in normal and cancer samples. **F** Boxplots showing the expressions of CSRP2BP in different stages of cervical cancers. **G** Scatter plot showing the correlation between the expressions of CSRP2BP and EMT scores. **H** The enrichment score (ES) distribution for genes positively or negatively correlated with CSRP2BP in EMT pathway

Histone acetylation has been demonstrated as one of the most important regulatory mechanisms of the EMT pathway. To systematically identify the histone acetylation-related genes that are potentially correlated with EMT, we first obtained 88 genes with histone acetyltransferase activity from GO. We next analysed the expression patterns of histone acetyltransferase genes in 128 cervical tissue specimens [19]. In total, 28 genes (e.g., ATF2, BRCA2, CSRP2BP and EP300) were up-regulated and 16 genes (e.g., CDY1, CHAT and CRAT) were down-regulated in cervical cancer (Fig. 1A). We ranked the genes based on R scores (see methods in detail) and identified the top 30% genes in cervical cancer (Fig. 1B). BRCA2 was ranked top one and emerging evidence have demonstrated that BRCA2 plays an important role in cervical cancer [27].

Next, we performed the SCC analysis and identified genes of which expressions were correlated with EMT scores in cervical cancer. In total, 19 genes and 26 genes that were negatively and positively correlated with EMT scores were identified (Fig. 1C). In particular, seven genes showed higher expression in cervical cancer and positively correlated with EMT scores, including CSRP2BP, LPCAT1, NAT14, CREBBP, MSL3, ATF2 and GNPNAT1 (Fig. 1D). We found that histone acetyltransferase CSRP2BP is a component of the ATAC chromatin remodelling complex containing HAT activity [28]. CSRP2BP is a co-activator for CRP2 to drive smooth muscle gene expression. Knockdown of CSRP2BP expression suppressed the migration of both A7r5 rat smooth muscle cells and primary human vascular smooth muscle cells (hVSMCs) [26]. However, the function of CSRP2BP in cervical cancer progression and metastasis is still unknown. Thus, we next analysed the expressions of CSRP2BP in cervical cancer in detail. We illustrated that CSRP2BP was highly expressed in cervical cancer compared to normal samples (Fig. 1E, *P* = *0.022*). In addition, CSRP2BP exhibited increased expression with CINIII and invasive cancer (Fig. 1F). Scatter plot showed that the expression of CSRP2BP was significantly correlated with EMT scores in cervical cancer (Fig. 1G, *R* = 0.34 and *P* = *7.5E-5*). Moreover, the GSEA found that genes correlated with CSRP2BP were significantly enriched in the EMT pathway (Fig. 1H, ES = 0.377 and *P* = *1.11E-8*). Together, these results suggest that CSRP2BP plays oncogenic roles in cervical cancer by potentially regulating EMT pathway.

### CSRP2BP is overexpressed in cervical cancer and correlated with a poor prognosis of cervical cancer patients

**Fig. 2:**
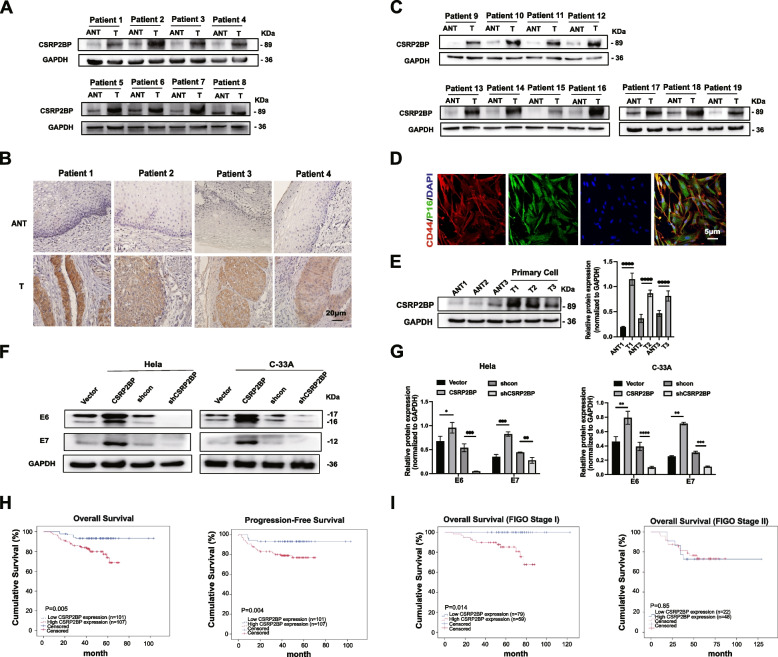
CSRP2BP overexpression correlates with a poor prognosis in cervical cancer patients. **A** and **C** Western blotting was performed to analyse the expression levels of CSRP2BP in 19 matched pairs of T and ANTs. GAPDH was the internal control. **B** IHC were used to analyze the specimens derived from human cervical cancer tissues (T) and adjacent noncancerous tissue samples (ANT) (*n* = 4, Scale bar 20 μm). **D** Cervical cancer primary cells were isolated from three cervical cancer tissues, and detected the expressions of CD44 (red) and P16 (green) by immunofluorescence staining. Nuclei were counterstained with DAPI (blue). (Scale bar 5 μm). **E** Western blotting was performed to analyze the expression levels of CSRP2BP in cervical cancer primary cells (T1, T2, T3) and ANTs (ANT1, ANT2, ANT3). **F**-**G** Western blotting was used to study the relationship between E6, E7 and CSRP2BP expression. GAPDH was used as loading control. **H** Kaplan–Meier overall survival curves (left panel), progression-free survival (right panel) and univariate analyses (log-rank) comparing cervical cancer patients with low (*n* = 101) and high (*n* = 107) CSRP2BP-expressing tumours. **I** Kaplan–Meier FIGO stage I overall survival curves (left panel), FIGO stage II overall survival curves (right panel) and univariate analyses (log-rank) comparing cervical cancer patients with low and high CSRP2BP-expression tumours. (*P < 0.05, **P < *0.01*, ***P < *0.001*, ****P < *0.0001*)

Previous studies have found that CSRP2BP is involved in cell differentiation, cell apoptosis and embryonic development [28, 29]. However, its function in tumorigenesis remains unclear. To investigate the role of CSRP2BP in cervical cancer, we performed Western blotting and IHC for the expression of CSRP2BP on human cervical tissues. We found that CSRP2BP was mainly expressed in carcinoma nests and expressed at significantly higher levels in cervical cancer tissues (T) than in adjacent noncancerous tissues (ANTs) (Fig. 2A-C). Cervical cancer primary cells (T1, T2, T3) were isolated from three cervical cancer tissues, and we found CSRP2BP expression was increased in primary cervical cancer cells compared with ANTs (Fig. 2D, E). These data indicated that the CSRP2BP expression was upregulated in cervical cancer.

To further validate the association of CSRP2BP expression with the prognosis of cervical cancer patients, specimens from a total of 208 patients who received surgery were analysed by IHC. According to the immunostaining scores of CSRP2BP, these patients were divided into high (score ≥ 6) or low/undetected (score ≤ 4) expression groups (Fig. S1). As shown in supplemental Table S5, high expression of CSRP2BP was associated with International Federation of Gynecology and Obstetrics (FIGO) stage (*P* < *0.001*), type of tumour growth (*P* < *0.001*), tumour size (*P* < *0.001*), stromal invasion (*P* < *0.001*), lymphovascular space invasion (LVSI) (*P* = *0.026*) and HPV16/18 infection (*P* < *0.001*). To find out the relationship between HPV16/18 infection and CSRP2BP, we tested the expression of E6/E7 in overexpressed/knockdown CSRP2BP cervical cancer cells. The results revealed that overexpression of CSRP2BP increased the E6/E7 expression and that knockdown CSRP2BP suppressed the E6/E7 expression (Fig. 2F, G). Consistent with our results, we found that CSRP2BP expression was higher in CINIII and cancer compared with normal tissues (Fig. 1F). The HPV 16/18 infection and development of the cervical lesion were therefore associated with an increased expression of CSRP2BP.

We next performed a Kaplan–Meier analysis to investigate the relationship between the expression of CSRP2BP and the survival of patients with cervical cancer. The median OS of patients with a high and low/no expression of CSRP2BP was 55 months (range, 8–88 months) and 62 months (range, 19–129 months) respectively (log-rank test χ2 = 8.014, *P* = *0.005*). The median PFS of patients with a high and low/no expression of CSRP2BP was 54 months (range, 1–88 months) and 60 months (range, 11–129 months) respectively (log-rank test χ2 = 8.319, *P* = *0.004*). These results revealed a clear negative correlation between the expression of CSRP2BP and PFS/OS of patients with cervical cancer (Fig. 2H). To analyse the effect of CSRP2BP on the OS of different FIGO stage cervical cancer patients, survival analysis was performed in FIGO I and II stage subgroups. Survival analysis revealed that CSRP2BP overexpression was associated with poor OS (*P* = *0.014*) in138 patients with stage I, but not with OS (*P* = *0.85*) in 70 patients with stage II (Fig. 2I). To further determine whether CSRP2BP served as an independent prognostic factor, PFS and OS were assessed using multivariate analysis. High CSRP2BP expression, LVSI and advanced FIGO stage were associated with both poor PFS and OS (Supplemental Table S6). Taken together, these data reveal a clear association between CSRP2BP expression and poor clinical outcome in cervical cancer patients.

### CSRP2BP enhances cervical cancer cell proliferation and growth in vitro and in vivo

**Fig. 3:**
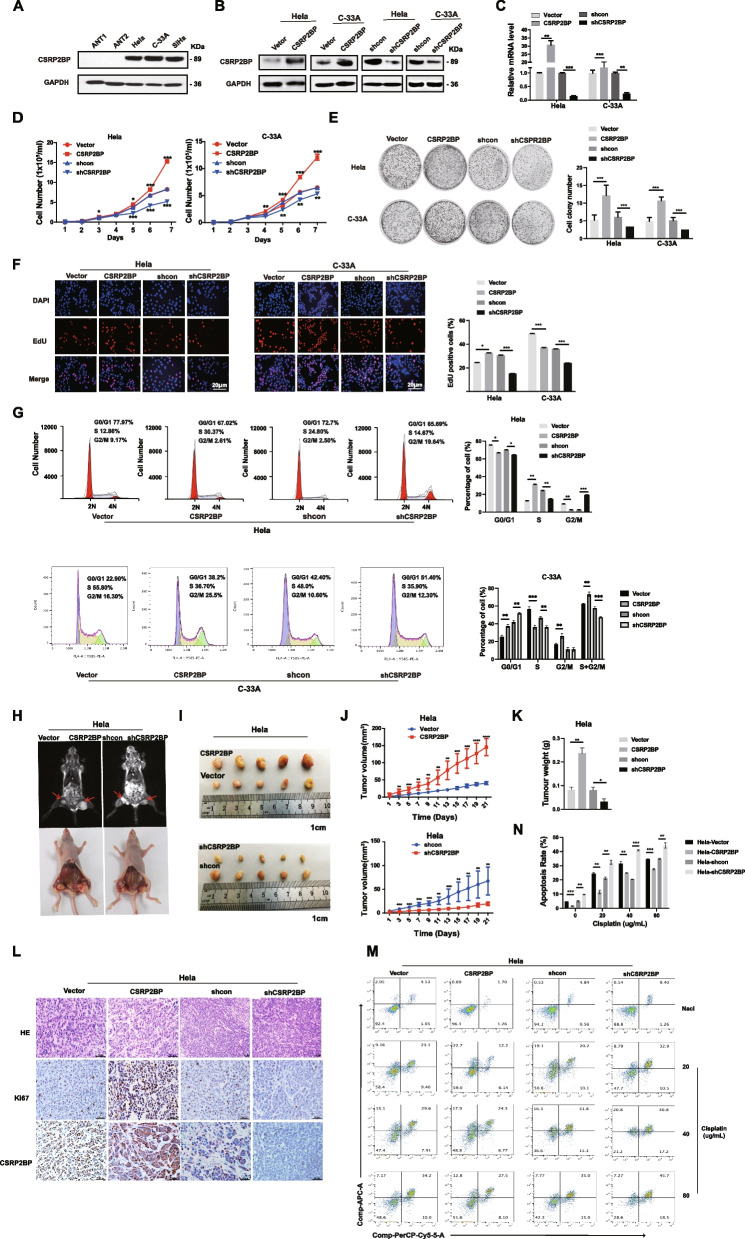
CSRP2BP enhances cervical cancer cell proliferation in vitro and in vivo. **A** CSRP2BP protein levels in three cervical cancer cell lines (Hela, SiHa and C-33A) and ANTs (ANT1 and ANT2) were examined by Western blotting. **B**, **C** Stable overexpression and knockdown of CSRP2BP in Hela and C-33A cell lines were evaluated by Western blotting and RT‒PCR. **D**-**E** The effect of CSRP2BP overexpression or knockdown on the proliferation of Hela and C-33A cells was examined by cell growth curve and colony formation assays. In cell growth curve, the relative cell number was expressed as the fold change relative to that on Day 0. The data are presented as the mean ± SD. Statistical significance was assessed by a two-tailed Student’s *t* test*.* **F** EdU assays were performed to evaluate the proportion of cells in cell cycle S-phase (*n* = 6, scale bar 20 µm). **G** Cell cycle flow cytometric analysis of CSRP2BP overexpressing/knockdown Hela/C-33A cells and vector control cells. **H** Generation of the xenograft model in BALB/c-nude mice. Hela-CSRP2BP, Hela-shCSRP2BP and the respective control cells were inoculated into BALB/c-nude mice (*n* = 5/group). Representative MRI images of the tumours (top). **I**-**K** Tumour volume and weight were significantly increased in Hela-CSRP2BP cells. Compared with that in the control group, volume of tumours derived from Hela-shCSRP2BP cells was significantly smaller. Data are shown as the mean ± SD (*n* = 5, scale bar 1 cm). **L** H&E staining of representative samples derived from mouse xenografts. The trends in CSRP2BP expression and Ki-67 staining by IHC were consistent (Scale bar 50 µm). **M**, **N** CSRP2BP enchances cervical cancer proliferation and induces chemoresistant. A Flow cytometry analysis of annexin V + /PI cells after the indicated cells were treated with cisplatin (20 µg/ml, 40 µg/ml and 80 µg/ml) for 24 h. Results were expressed as percentages of apoptosis cells. All experiments were repeated at least 3 times independently with each carried out in triplicate. (*P < 0.05, **P < 0.01, ***P < 0.001, ****P < 0.0001)

To evaluate the functions of CSRP2BP, Western blotting was first used to evaluate the expression of CSRP2BP in cervical cancer cell lines (Hela, SiHa and C-33A) and ANTs. We found that the protein levels of CSRP2BP were prominently increased in cervical cancer cell lines (Fig. 3A), suggesting its potential tumorigenic role in cervical cancer. To test whether CSRP2BP was involved in cervical cancer cell growth and proliferation, we assessed the proliferation potential of CSRP2BP-overexpressing Hela/C-33A cells (Hela-CSRP2BP, C-33A-CSRP2BP) as well as CSRP2BP-knockdown Hela/C-33A cells (Hela-shCSRP2BP, C-33A-shCSRP2BP) (Fig. 3B, C). Cell growth curve and colony formation assays showed that CSRP2BP overexpression significantly increased the proliferation of Hela/C-33A cells, whereas knockdown of CSRP2BP suppressed the proliferation of Hela/C-33A cells (Fig. 3D, E). Furthermore, EdU staining and flow cytometry assays demonstrated that the proportion of S-phase cells significantly increased with the overexpression of CSRP2BP, whereas knockdown of CSRP2BP had the opposite effect in Hela cells. Meantime, the proportion of overexpressing CSRP2BP C-33A cells decreased in the S phase and the cell cycle was blocked in S + G2/M phase significantly. The results suggested that overexpression of CSRP2BP promoted the proliferation of C-33A cells (Fig. 3F, G). Taken together, our data suggest that CSRP2BP accelerates the proliferation of cervical cancer cells in vitro.

To further confirm the functions of CSRP2BP, we examined the effect of CSRP2BP on the tumorigenicity of cervical cancer in vivo by using a subcutaneous xenograft model. As shown in Fig. 3H and I, MRI experiments and isolated tumour specimens revealed that CSRP2BP overexpression increased tumour growth and weight, while knockdown of CSRP2BP obtained the opposite results. Meantime, CSRP2BP promoted Hela cell growth in vivo (Fig. 3J, K). IHC results further illustrated that Ki-67 was increased in CSRP2BP-overexpressing tumours, while Ki-67 was decreased in CSRP2BP-knockdown tumours (Fig. 3L). These results demonstrate that CSRP2BP induces the growth and proliferation of cervical cancer both in vitro and in vivo.

Cisplatin is one of the most commonly used chemotherapeutic drugs in the treatment of cervical cancer [30]. We assessed the chemotherapy sensitivity of cervical cancer cells. Hela-vector and Hela-CSRP2BP cells were stained with annexin V + and PI. The results showed that the overexpression of CSRP2BP induced apoptosis resistance in cisplatin-treated cervical cancer cells (Fig. 3M, N). Our findings revealed that the cell survival rate increased and that the sensitivity of cervical cancer cells to cisplatin decreased in a dose-dependent manner with the overexpression of CSRP2BP.

### CSRP2BP drives cervical cancer invasion and metastasis

**Fig. 4:**
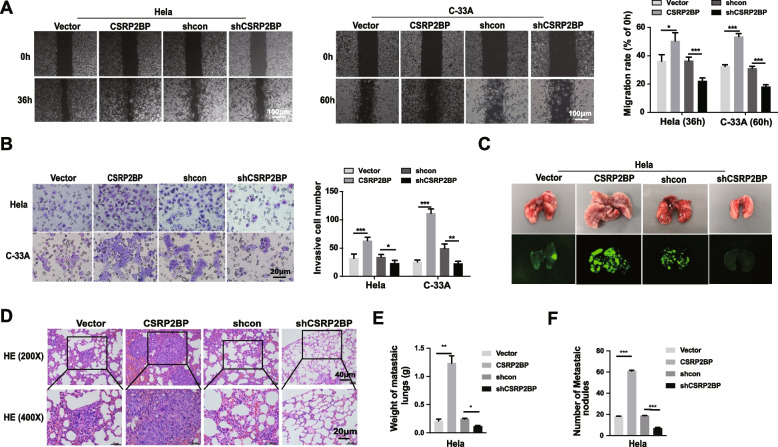
CSRP2BP promotes cervical cancer metastasis in vitro and in vivo. **A** Wound healing assays indicated that the migration rate increased in CSRP2BP-overexpressing cells and decreased in CSRP2BP knockdown cells. The imgration rates are expressed as the area percentage at 0 h (scale bar 100 µm). Data are shown as the mean ± SD (*n* = 6). **B** Transwell assays showed that the invasion rates increased in CSRP2BP-overexpressing cells and decreased in CSRP2BP knockdown cells (scale bar 20 µm). Data are shown as the mean ± SD (*n* = 3). **C** Hela-CSRP2BP, Hela-shCSRP2BP and the respective control cells were injected into BALB/c-nude mice through the tail vein (*n* = 5). Representative photos of lungs isolated from mice. Green fluorescence was derived from EGFP of cells using a chemiluminescent imaging system *in* vitro imaging. **D** H&E staining of representative samples derived from mice lungs (scale bars, 40 µm and 20 µm, respectively). **E**, **F** Lung weight and the number of metastatic nodules were significantly increased in Hela-CSRP2BP cells and decreased in Hela-shCSRP2BP cells. Data are shown as the mean ± SD. (*P < 0.05, **P < 0.01, ***P < 0.001)

Cancer metastasis is typically characterized by tumour cell properties of increased migration and invasion [31]. We next investigated whether CSRP2BP impacts cell invasion and migration in cervical cancer. As shown in Fig. 4A and B, CSRP2BP overexpression greatly increased the migration and invasion of Hela/C-33A cells, while CSRP2BP knockdown cells showed a reduced metastatic capacity in vitro. To elucidate whether CSRP2BP promoted cervical cancer metastasis in vivo, we injected CSRP2BP-overexpressing and CSRP2BP-knockdown Hela cells through the tail vein of mice to establish a pulmonary metastasis model in BALB/c nude mice. Six weeks later, the lungs were collected, and micro-metastatic foci were counted. In line with our in vitro results, CSRP2BP overexpression showed more lung metastatic foci and a higher metastasis incidence, as determined by fluorescence and H&E staining (Fig. 4C, D). The tumour weight and number induced by the injection of Hela-CSRP2BP cells and Hela-shCSRP2BP was respectively significantly larger and smaller than control cells (Fig. 4E, F). Taken together, these results indicate that CSRP2BP induces cervical cancer cell invasion and metastasis both in vitro and in vivo.

### CSRP2BP activates EMT signals and increases metastasis in cervical cancer cells

**Fig. 5:**
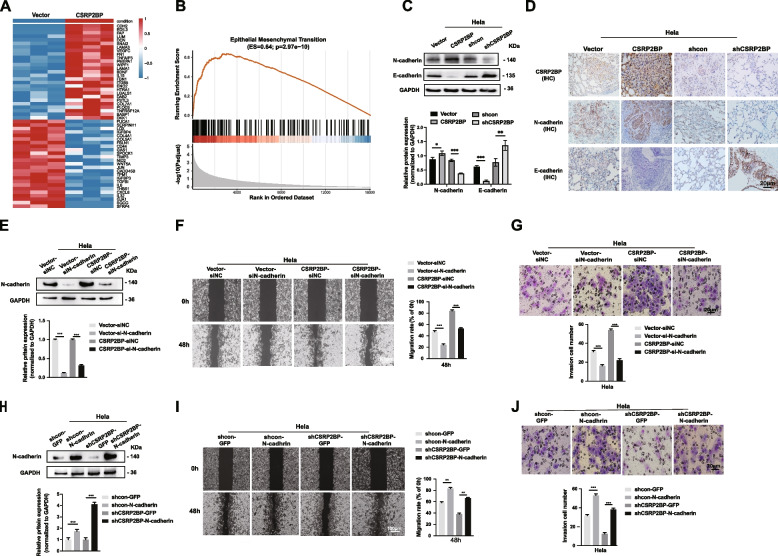
CSRP2BP activates N-cadherin expression to enhance cervical cancer cell metastasis. **A** Heatmap representation of the top-ranked genes upregulated and downregulated in Hela-CSRP2BP cells compared with the control group. **B** The GSEA plot revealed that CSRP2BP expression levels were positively correlated with EMT. **C** The protein expression levels of N-cadherin and E-cadherin in Hela-CSRP2BP and Hela-shCSRP2BP cells were detected by Western blotting. **D** The trend in the expression of CSRP2BP, N-cadherin and E-cadherin by IHC is consistent in metastatic tumors of mice lungs. (Scale bar 20 µm). **E** Hela-CSRP2BP cells and control cells were treated with siRNA targeting N-cadherin for 48 h. N-cadherin silencing in Hela-CSRP2BP cells was confirmed by Western blotting. **F** Wound healing and Transwell assays **G** indicated that the migration and invasion rates decreased in Hela-CSRP2BP-siN-cadherin cells. **H** Hela-shCSRP2BP and control cells were transfected with pSin-N-cadherin and control plasmids. N-cadherin overexpression in Hela-shCSRP2BP cells was confirmed by Western blotting. **I** Wound healing and Transwell assays **J** indicated that the migration and invasion rates increased in Hela-shCSRP2BP-N-cadherin cells. Data are shown as the mean ± SD (*n* = 6). Data are shown as the mean ± SD (*n* = 6). (*P<0.05, **P < 0.01, ***P < *0.001*)

Since histone acetylation is a ubiquitous hallmark of transcriptional activity [32], we conducted RNA-Seq in CSRP2BP-overexpressing and control Hela cells for a full transcriptome analysis to monitor potential downstream targets of CSRP2BP in cervical cancer metastasis. Compared with the control groups, 591 genes were upregulated (≥ twofold) in Hela-CSRP2BP cells (Fig. S2A). GO analysis showed that most of these genes were involved in cell proliferation and adhesion, consistent with our study (Fig. S2B). Kyoto Encyclopedia of Genes and Genomes (KEGG) pathway enrichment analysis revealed that the most significantly activated pathway was the PI3K/AKT signalling pathway (Fig. S2C, D). Intriguingly, N-cadherin (also known as CDH2) was found among the genes that were the most upregulated in each of the Hela-CSRP2BP cells, and this gene is associated with EMT signalling and cancer metastasis (Fig. 5A and Fig. S2E). The GSEA analysis revealed that CSRP2BP expression levels were positively correlated with EMT (Fig. 5B, ES = 0.64, *P* = *2.97e-10*). These results were consistent with our finding that CSRP2BP showed high expression in cervical cancer and positively correlated with EMT (Fig. 1C, D). Therefore, we hypothesized that N-cadherin might be an important downstream transcriptional target involved in CSRP2BP-mediated cervical cancer EMT and metastasis.

To test this hypothesis, we examined the expression of N-cadherin and E-cadherin by Western blotting and RT‒PCR. As shown in Fig. 5C and Fig. S2F, a significant increase in N-cadherin protein expression and a reduction in E-cadherin protein expression were observed in Hela-CSRP2BP cell extracts. Subsequent IHC also revealed that the expression of N-cadherin was significantly increased in Hela-CSRP2BP xenograft tumours, while the expression of E-cadherin was decreased (Fig. 5D). This led us to further examine a variety of EMT and invasion markers. Of note, MMP-2 and MMP-9 showed increased mRNA and protein levels in CSRP2BP-overexpressing cells, whereas CSRP2BP-knockdown cells displayed the opposite results (Fig. S2G, H). To verify that N-cadherin was, at least in part, under the control of CSRP2BP during EMT, we used small interfering RNA (siRNA) to knockdown N-cadherin in Hela-CSRP2BP cells (Hela-CSRP2BP-siN-cadherin, Fig. 5E). Depletion of N-cadherin reduced cell metastasis and invasion in vitro (Fig. 5F, G). Further study demonstrated that N-cadherin overexpression was able to increase the metastasis and invasion capacity of Hela-shCSRP2BP cells (Hela-shCSRP2BP-N-cadherin, Fig. 5H-J). These data strongly demonstrate a critical role of N-cadherin in CSRP2BP-induced EMT signalling in the metastasis process.

### Identification of SMAD4 as a CSRP2BP binding partner that induces N-cadherin transcription

**Fig. 6:**
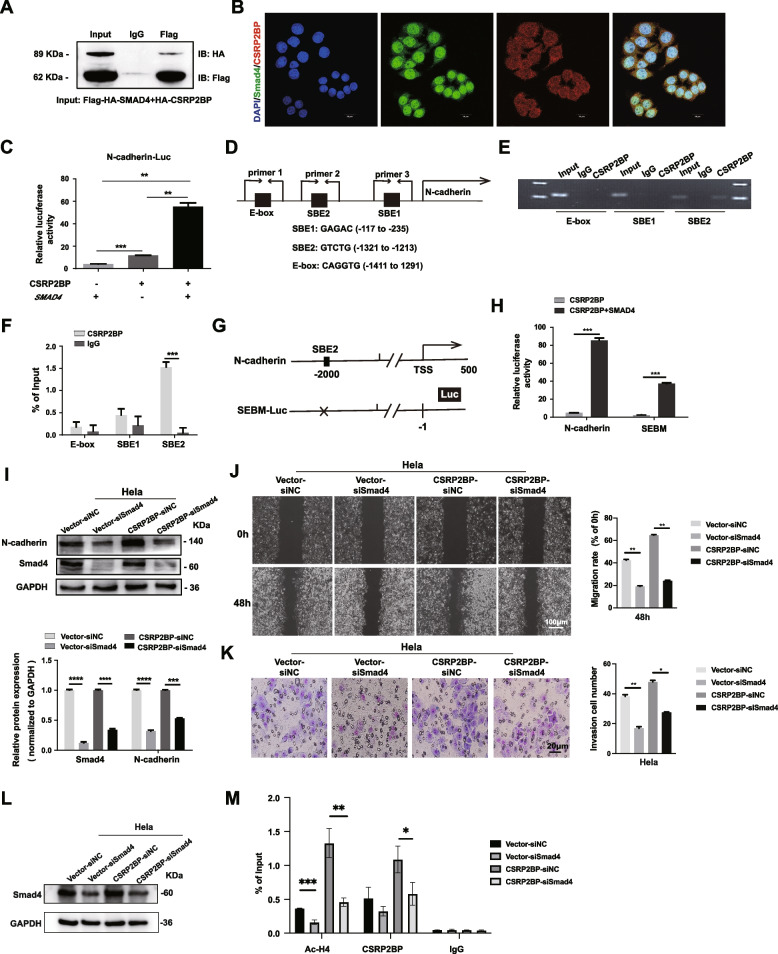
CSRP2BP regulates N-cadherin transcription by recruiting SMAD4 in cervical cancer. **A** The interaction between CSRP2BP and SMAD4 was tested by Co-IP assay with anti-Flag antibody and control normal IgG. Western blotting was performed with an anti-HA antibody. **B** Subcellular co-expression of CSRP2BP (red) and SMAD4 (green) in Hela cells was detected by immunofluorescence staining. Nuclei were counterstained with DAPI (blue). (Scale bar, 20 µm). **C** A luciferase reporter gene assay was used to evaluate SMAD4 promoting N-cadherin promoter transcription (*n* = 3). **D** Position of the SMAD4-binding site in the N-cadherin promoter. **E** CSRP2BP occupies the E-box, SBE1 and SBE2 of the N-cadherin promoter region, as measured by ChIP-PCR assay. **F** CSRP2BP levels in the N-cadherin promoter region were analysed by ChIP‒QPCR assay. IgG was used as a negative control. **G** The wild type and mutated constructs of the *N-cadherin* promoter-luciferase reporter (*N-cadherin*-Luc). SEBM-Luc had mutated SEB2. **H** Cell transfection assays. CSRP2BP/SMAD4 complex showed much less increased the activities of SBEM-Luc versus *N-cadherin*-Luc in Hela cells. **I** Hela-CSRP2BP cells and control cells were pre-treated with SMAD4 siRNA for 48 h and then detected by Western blotting. **J** Wound healing assays and Transwell assays **K** revealed that the migration and invasion rates decreased in Hela-CSRP2BP-siSMAD4 cells. Data are shown as the mean ± SD (*n* = 6). **L** Hela-CSRP2BP cells and control cells were pretreated with SMAD4 siRNA for 48 h and then detected SMAD4 expression by Western blotting. **M** CSRP2BP and H4Ac levels in the N-cadherin promoter region were analysed by ChIP‒QPCR assay upon SMAD4 knockdown in Hela-CSRP2BP cells. IgG was used as a negative control. (*P < 0.05, **P < 0.01, ***P < 0.001, ****P<0.0001)

As a transcriptional coactivator, CSRP2BP does not bind DNA itself; it must be recruited to chromatin via protein‒protein interactions with transcription factors to regulate gene expression [26]. Since studies reported that the transcription factors SMAD3 and 4 could bind to the promoter region of N-cadherin to stimulate its transcription and participate in the EMT process [33, 34], we hypothesized that CSRP2BP might associate with SMAD4 or SMAD3 to promote N-cadherin transcription. We cotransfected plasmids expressing flag-HA-tagged SMAD3 or flag-HA-tagged SMAD4 in Hela cells with HA-tagged CSRP2BP, and the Co-IP results indicated that CSRP2BP interacted with SMAD4 but not with SMAD3 (Fig. 6A and Fig. S3). Next, we determined the subcellular localization of CSRP2BP and SMAD4 by performing immunofluorescence. As shown in Fig. 6B, endogenous CSRP2BP and SMAD4 were expressed in both the nucleus and cytoplasm of cervical cancer cells. To further explore the function of the CSRP2BP/SMAD4 complex, we conducted a luciferase reporter gene assay using a pGL-3-N-cadherin promoter plasmid with CSRP2BP and SMAD4 expression plasmids. CSRP2BP significantly increased the luciferase activity in the pGL-3-N-cadherin promoter when SMAD4 was present (Fig. 6C).

Next, we explored whether CSRP2BP was recruited to the N-cadherin promoter by SMAD4 in Hela cells. The N-cadherin promoter contains two SMAD binding elements (SBEs), -117 to -235 and -1321 to -1213, in the ~ 2 Kb promoter region [35–37]. We then conducted ChIP-QPCR with anti-CSRP2BP or control IgG antibodies to test whether these two sites are involved in the binding of CSRP2BP and SMAD4 to the N-cadherin promoter. We also tested the nearby E-box site (-1411 to -1291) as a control. ChIP-QPCR results showed that CSRP2BP levels were high surrounding the SBE2 site, while no obvious binding around SBE1 and the E-box site was observed in Hela cells (Fig. 6D-F). Deletion of SBE2 in N-cadherin-Luc (SEBM-Luc) reporter significantly reduced SMAD4/CSRP2BP complex-increased N-cadherin promoter activity, supporting the idea that SMAD4 recruits CSRP2BP specifically to the SBE2 site of the N-cadherin promoter in cervical cancer cells (Fig. 6G, H).

To probe whether SMAD4 was the key factor by which CSRP2BP activated N-cadherin expression in cervical cancer cells, we used siRNA to knockdown SMAD4 in Hela-CSRP2BP cells and detected changes in SMAD4 and N-cadherin expression. As expected, SMAD4 knockdown decreased the expression of N-cadherin (Fig. 6I). Furthermore, Transwell and cell migration assays revealed that knockdown of SMAD4 significantly inhibited the invasion and metastasis abilities of CSRP2BP-overexpressing cells (Fig. 6J, K). Next, we knocked down SMAD4 expression by using siRNA and performed ChIP-QPCR to verify that CSRP2BP promotes histone acetylation by binding to SMAD4 on the SBE2 of N-cadherin promoter. As shown in Fig. 6L-M, SMAD4 knockdown significantly reduced the binding of CSRP2BP to Ac-H4. Meantime, the binding of CSRP2BP to the N-cadherin promoter region SBE2 was also reduced. In summary, CSRP2BP stimulates N-cadherin transcription by cooperating with SMAD4 to bind to the N-cadherin promoter.

### CSRP2BP promotes N-cadherin expression partly by acetylating H4

**Fig. 7:**
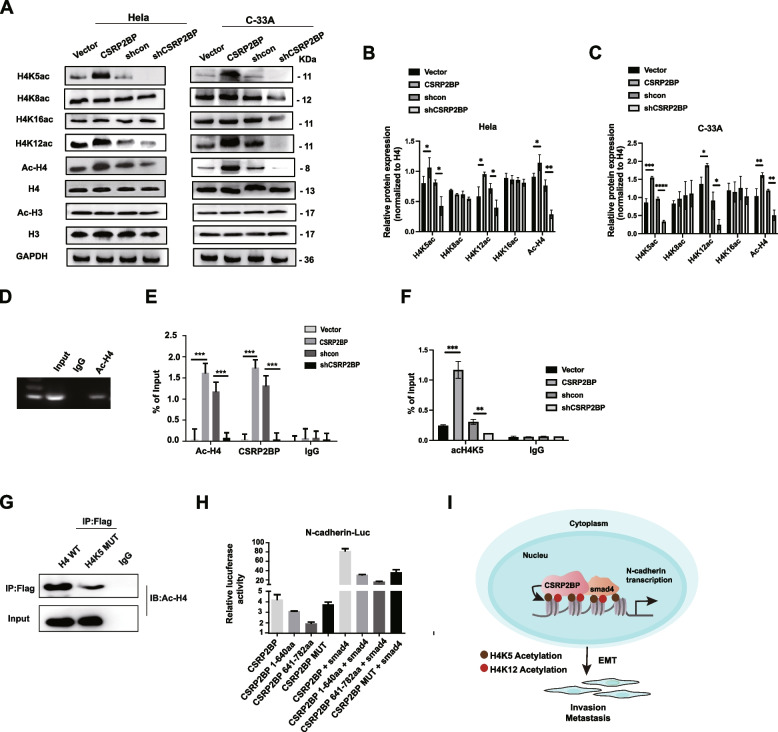
CSRP2BP activates the N-cadherin promoter by acetylating histone H4. **A**-**C** Acetylation of H4, H4K5, H4K8, H4K12 and H4K16 was analysed by Western blotting. Total H4 was used as a loading control. The quantification of Western Blot was performed by ImageJ software. All data are presented as mean ± SD of 3 independent experiments (*n* = 3). **D** Histone H4 acetylation levels in the N-cadherin promoter region were determined by ChIP‒PCR assay. IgG was used as the negative control. **E** ChIP‒QPCR assays were performed using antibodies against Ac-H4 and CSRP2BP in Hela-CSRP2BP cells, Hela-shCSRP2BP cells and their respective controls. IgG was used as a negative control. **F** H4K5ac levels in the N-cadherin promoter region were analysed by ChIP‒QPCR assay. IgG was used as a negative control. **G** Immunoprecipitation assay was performed to evaluate that CSRP2BP promoted H4 acetylation through K5 site. Flag-HA-tagged H4 plasmid (H4-WT) and flag-HA-tagged H4K5 mutant plasmid (H4K5 MUT) was respectively transfected into Hela-CSRP2BP cells. IgG was used as the negative control. The level of H4Ac was detected by Western blotting. **H** A luciferase reporter assay was used to detect the effect of CSRP2BP HAT domain mutation (CSRP2BP MUT) on its binding to SMAD4 to promote N-cadherin promoter transcription in Hela cells (*n* = 3). **I** Graphic model of CSRP2BP as discussed in the text. In cervical cancer, CSRP2BP causes a significant increase in H4 acetylation at lysine (**K**) sites 5 and 12 (K5 and K12). Moreover, CSRP2BP promoted N-cadherin transcription by binding the transcription factor SMAD4 to the N-cadherin promoter and enhancing cervical cancer EMT and metastasis. DNA (grey line); acetylated histone sites (histone 4 lysine 5, brown ball; histone 4 lysine 12, red ball). All data are mean ± SD of 3 independent experiments performed in triplicate. (*P < 0.05, **P < 0.01, ***P < 0.001, ****P < 0.0001)

Histone acetylation makes a great contribution to EMT, and CSRP2BP is one of the important HATs [38, 39]. We previously reported that CSRP2BP acetylated H3 and H4 [26]. In addition, Drosophila CSRP2BP was shown to exhibit HAT activity specific for H4 [29]. Thus, to verify whether CSRP2BP promoted N-cadherin expression via histone acetylation in cervical cancer cells, we subsequently checked the levels of histone H3 acetylation (H3Ac) and histone H4 acetylation (H4Ac) in CSRP2BP-overexpressing or CSRP2BP-silenced Hela/C-33A cells. As shown in Fig. 7A-C, CSRP2BP overexpression significantly increased the histone H4 acetylation level at lysine sites 5 and 12 in Hela and C-33A cells. No other checked sites of histone H4 (lysine sites 8 and 16) or histone H3 acetylation were changed.

Next, to verify whether CSRP2BP regulates N-cadherin transcription by histone acetylation, we examined H4 acetylation levels on the N-cadherin promoter by ChIP assay and found that CSRP2BP overexpression led to an increased level of H4Ac on the N-cadherin promoter in Hela cells (Fig. 7D, E). Further, we examined H4K5 acetylation levels on the N-cadherin promoter by ChIP assay. As shwon in Fig. 7F, CSRP2BP overexpression led to an increased level of H4K5Ac on the N-cadherin promoter in Hela cells. In addition, we generated flag-HA-tagged H4 expression plasmid and flag-HA-tagged H4K5 mutant (lacking the K5 site (H4K5 MUT)) plasmids, and immunoprecipitation assay was used to verify that CSRP2BP promoted H4 acetylation through K5 site. The results revealed that H4K5 MUT led to a decreased expression of H4Ac in Hela-CSRP2BP cells (Fig. 7G). Our previous study showed that the HAT domains of CSRP2BP in mice are located at amino acids 708–710 [26]. Since the HAT domains are highly conserved, we therefore generated a human CSRP2BP HAT mutant by deleting four amino acids within the HAT domain (711–714 aa deleted (CSRP2BP MUT)) and assayed the effects of CSRP2BP MUT on co-activation of the N-cadherin promoter by SMAD4. CSRP2BP MUT marginally reduced the transcription of N-cadherin compared to that of wild-type CSRP2BP (Fig. 7H). All these results indicate that CSRP2BP activates N-cadherin transcription primarily through its histone acetylation activity.

## Discussion

The metastasis of cervical cancer is closely correlated with a poor prognosis and low 5-year survival rates in cervical cancer patients [40]. Therefore, there is an urgent need to unravel the precise mechanisms underpinning the metastasis of cervical cancer. EMT is an important process during metastasis, and histone acetylation is one of the most important regulators of cervical cancer metastasis. In the present study, we analysed cervical tissue gene expression data and identified the histone acetylation-related genes that potentially correlated with EMT. We found that the histone acetyltransferase CSRP2BP was highly expressed in cervical cancer and positively correlated with EMT scores. In addition, CSRP2BP exhibited increased expression with CINIII and invasive cancer, indicative of the association of increased CSRP2BP expression with the development of cervical lesion. Mechanistically, we found that 1) CSRP2BP promoted cervical cancer EMT and metastasis by upregulating N-cadherin, and 2) CSRP2BP increased N-cadherin expression by acetylating H4K5 and H4K12 in the promotor region of N-cadherin through cooperation with Smad4 (Fig. 7I). These results provided strong evidence that CSRP2BP was an important histone acetyltransferase and oncogenic factor in EMT and metastasis of cervical cancer.

Acetylation is one of the posttranscriptional modifications (PTMs) influencing various aspects of protein biology [41]. Histone acetylation and deacetylation is mediated by HATs and HDACs, and its balance is required for normal function of a variety of cells and tissues. Indeed, accumulating evidence has shown that aberrant histone acetylation contributes to pathogenesis of many diseases, such as cancer, chronic inflammation and diabetes [42, 43]. HATs catalyse acetylation associated with gene transcription, and a growing number of studies have highlighted the role of HAT dysfunction in the initiation and development of various cancers, such as breast and liver cancers [44]. However, the precise mechanisms underlying how HATs regulate the progression and metastasis of cervical cancer are still elusive. The histone acetyltransferase CSRP2BP is a co-activator for CRP2, and our previous study revealed that CSRP2BP was a driver of smooth muscle gene expression [26]. However, the role of CSRP2BP in the metastasis of cervical cancer is poorly understood. In the present study, we found that 1) the CSRP2BP expression was significantly increased in clinical cervical cancer tissues and cell lines, 2) CSRP2BP overexpression promoted tumour growth and metastasis in both vitro and vivo, 3) CSRP2BP knockdown significantly inhibited tumour growth and metastasis, and 4) clinically, elevated expression of CSRP2BP was significantly correlated with decreased overall survival rates. These findings support the premise that CSRP2BP is an oncogenic factor and potentially a biomarker for the long-term survival of cervical cancer patients.

It is important to note that a significant association between the CSRP2BP expression and the clinicopathological characteristics of cervical cancer patients was observed, including FIGO stage and HPV16/18 infection. More importantly, overexpression of CSRP2BP induced but knockdown of CSRP2BP suppressed E6/E7 expression. Thus, it was highly likely that CSRP2BP participated in the process of HPV infection and the development of the cervical lesion, although the underlying mechanisms need further investigation. On the other hand, CSRP2BP overexpression was associated with poor OS in 138 patients with stage I, but not with OS in 70 patients with stage II. Gene expression analysis showed that the expression of CSRP2BP was higher in cervical cancer and CINIII compared with normal tissues. These findings suggested that CSRP2BP may play a vital role in the carcinogenesis and progression of cervical cancer. Further studies on the potential role of CSRP2BP in cervical basement membrane discontinuity (breaks or absence) would be of great interest.

We further observed that the CSRP2BP overexpression decreased the sensitivity of cervical cancer cells to cisplatin in a dose-dependent manner, suggesting the involvement of CSRP2BP in the chemotherapy resistance of cervical cancer. Based on the above findings, we speculated that CSRP2BP might serve as a novel biomarker and a potential therapeutic target for cervical cancer treatment.

EMT is a vital process contributing to cervical cancer progression, invasion and metastasis and characterized by the loss of epithelial markers, such as E-cadherin, and the gain of mesenchymal markers, such as N-cadherin and vimentin [45]. The activation of EMT, especially through the upregulation of N-cadherin, have been shown to make a great contribution to cancer metastasis, and interestingly, HATs play a vital regulatory role in EMT [46]. However, whether CSRP2BP participates in the regulation of the EMT process in cervical cancer is still unknown. Here, we revealed that N-cadherin expression was significantly increased in CSRP2BP-overexpressing Hela cells as analysed by RNA-seq. KEGG analysis also showed that EMT-related signalling was activated in CSRP2BP-overexpressing cells. Moreover, depletion of N-cadherin compromised the effects of overexpressed CSRP2BP on Hela cells. Thus, CSRP2BP overexpression increased N-cadherin expression in both vitro and vivo and promoted cervical cancer metastasis at least in part through upregulating N-cadherin. Moreover, the RNA-seq results revealed many other genes associated with cell adhesion were also significantly changed in CSRP2BP-overexpressing Hela cells. Future studies should be directed to probe the potential roles of these genes individually and collectively in cervical cancer progression and metastasis.

Our previous study showed that CSRP2BP strongly acetylated H3 and H4 [26]. However, whether CSRP2BP-induced acetylation of H3 and/or H4 is implicated in its upregulation of N-cadherin expression and promotion of cervical cancer carcinogenesis is still unknown. In the present study, we found that CSRP2BP overexpression significantly increased acetylation of H4 lysine 5 and 12 by binding to the SEB2 region of the promotor of N-cadherin. Furthermore, the CSRP2BP HAT domain mutant (711–714 aa deleted), which exhibited reduced HAT ability, was incapable of increasing N-cadherin expression with SMAD4, as seen for wild-type CSRP2BP, suggesting that CSRP2BP promoted N-cadherin transcription primarily by acetylating histones. A previous study demonstrated that HPV oncoprotein E7, which plays a central role in cervical carcinogenesis, promoted E7-expressing cell proliferation through acetylating H3K9 in the promoter of E2F1 mediated by the HAT GCN5, suggesting that H3 acetylation also contributes to cervical carcinogenesis [18]. Intriguingly, we found that CSRP2BP did not affect the acetylation level of H3 in cervical cancer cells. Thus, we speculated that 1) acetylation of H3 and H4 by different HATs may have differential physiological/pathological roles, and 2) acetylation of H3 and/or H4 by CSRP2BP is context dependent. Hence, investigating the regulatory networks underlying H3 and H4 acetylation by CSRP2BP in a variety of cell types and disease models is of great interest.

SMAD4 is one of the essential transcriptional regulators of EMT-associated genes including N-cadherin and also plays an essential role in the progression of cervical cancer [47–49]. Given the regulation of N-cadherin expression by CSRP2BP in cervical cancer, we hypothesized that CSRP2BP mediates the N-cadherin expression through SMAD4. Indeed, we found that CSRP2BP interacted with SMAD4 and that SMAD4 was a key transcription factor in CSRP2BP-mediated N-cadherin transcription. Previous findings showed that CSRP2BP mediates target gene expression through some transcription factors such as SRF [26]. Based on these findings, we reckoned that different transcription factors are important components for CSRP2BP-mediated functional networks, which are presumably context-dependent. Whether the CSRP2BP/SMAD4 complex coordinates with other factors to control specific H4 acetylation and EMT-associated gene expression in cervical cancer progression and metastasis awaits further investigation.

## Conlusions

We report here the critical role of CSRP2BP in EMT and metastasis of cervical cancer. Increased CSRP2BP expression is closely correlated with a poor prognosis in cervical cancer patients. Mechanistically, CSRP2BP promotes the EMT process partly by increasing H4 acetylation via cooperation with SMAD4, resulting in the upregulation of N-cadherin and finally promoting the invasion, migration and chemoresistance of cervical cancer cells in vitro and the metastasis of cervical cancer in vivo. We therefore conclude that CSRP2BP can be a clinical prognostic hallmark for cervical cancer patients and a potential therapeutic target for patients with cervical cancer metastasis.

### Supplementary Information

**Additional file 1: Supplemental Table S1.** Primers for plasmid construction. **Supplemental Table S2.** Antibodies used in this study. **Supplemental Table S3.** Primers for Real-time PCR. **Supplemental Table S4.** Primer for ChIP-PCR. **Supplemental Table S5.** Correlation between CSRP2BP expression and clinicopathologic features of cervical cancer (*N* = 208). **Supplemental Table S6.** Multivariate analysis of prognostic factors for cervical cancer patients. **Supplemental Table S7.** Laboratory apparatus used in this study

**Additional file 2: Supplemental Figure S1.** Expression of CSRP2BP in cervical cancer. Representative images of IHC staining for CSRP2BP in paraffin embedded sections of cervical cancer patients.

**Additional file 3: Supplemental Figure S2.** CSRP2BP induces EMT-related signals in Hela cells. (A) Heatmap representation of up-regulated and down-regulated genes in Hela-CSRP2BP cells compare with control group. (B) GO analysis of top 20 biological functions of up-regulated genes in Hela-CSRP2BP cells. (C) KEGG pathways enriched by the selected up-regulated genes. (D) The protein expression levels of the PI3K/AKT signalling pathway were detected in Hela-CSRP2BP cells, Hela-shCSRP2BP and the respective control cells by Western blotting. (E) Volcano Plots of up-regulated and down-regulated genes in Hela-CSRP2BP cells compare with control group. (F) The mRNA expression of N-cadherin and E-cadherin in Hela-CSRP2BP and Hela-shCSRP2BP cells were measured by RT-PCR. Statistical analysis was shown as mean SD (*n* = 3). (G) The protein expression levels of EMT-related markers were measured by Western blotting. (H) The mRNA expression levels of EMT-related markers were detected by RT-PCR. Statistical analysis is shown as mean ± SD (*n* = 3). (*P < 0.05, **P < 0.01, ***P < 0.001).

**Additional file 4: Supplemental Figure S3.** CSRP2BP does not bind to SMAD3 in Hela cells. The interaction between HA-tagged CSRP2BP and HA & flag-tagged SMAD3 was tested by Co-IP assay with anti-flag antibody or control normal IgG. Western blotting was stained with anti-HA antibody.

## Data Availability

All data supporting this study are available within this article and supplementary Information file. The dataset used and/or analyzed during the current study are available from the corresponding author on reasonable request.

## Abbreviations

EMT: Epithelial-mesenchymal transition
HATs: Histone acetyltransferases
HDACs: Histone deacetylases
KAT14: Lysine (K) acetyltransferase 14
KO: Knockout
hVSMCs: Human vascular smooth muscle cells
T: Tissue specimens
ANT: Adjacent noncancerous tissue
cDNA: Complementary DNA
PBS: Phosphate buffered saline
MRI: Magnetic resonance imaging
ChIP: Chromatin immunoprecipitation
Co-IP: Coimmunoprecipitation
IHC: Immunohistochemistry
FIGO: Federation International of Gynecology and Obstetrics
LVSI: Lymphovascular space invasion
OS: Overall survival
PFS: Progression-free survival
GO: Gene ontology
KEGG: Kyoto Encyclopedia of Genes and Genomes
siRNA: Small interfering RNA
H3Ac: Histone H3 acetylation
H4Ac: Histone H4 acetylation
CSRP2BP MUT: CSRP2BP HAT mutant
PTMs: The posttranscriptional modifications
