# Supplementary material for: Histone acetyltransferase CSRP2BP promotes the epithelial–mesenchymal transition and metastasis of cervical cancer cells by activating N-cadherin
Source: J Exp Clin Cancer Res. 2023 Oct 17;42:268. doi: 10.1186/s13046-023-02839-2 (PMC10580587; doi:10.1186/s13046-023-02839-2)
Supplement: Supplementary file 1 — Additional file 1: Supplemental Table S1. Primers for plasmid construction. Supplemental Table S2. Antibodies used in this study. Supplemental Table S3. Primers for Real-time PCR. Supplemental Table S4. Primer for ChIP-PCR. Supplemental Table S5. Correlation between CSRP2BP expression and clinicopathologic features of cervical cancer (N = 208). Supplemental Table S6. Multivariate analysis of prognostic factors for cervical cancer patients. Supplemental Table S7. Laboratory apparatus used in this study [file 13046_2023_2839_MOESM1_ESM.docx]

**Supplemental Materials**

**Supplemental Table S1. Primers for plasmid construction**

| **Name** | **Sequences** | **Companies** |
| --- | --- | --- |
| **shRNA-CSRP2BP** |  |  |
| CSRP2BP-shRNAi#1: | 5’-ATAACAGTAATCGAGAGGTGC-3’ | GIEE Shanghai |
| CSRP2BP-shRNAi#2: | 5’-AAGATCCGAGAGGTATAAGGG-3’ | GIEE Shanghai |
| **CSRP2BP-HAT-KOD** |  |  |
| Forward | 5’-ATTGCAACTTTCATGATCTATCATC -3’ | IGE GuangZhou |
| Reverse | 5’-CCATTCAGGGTGGACGAACAGAAAT-3’ | IGE GuangZhou |
| **CDH2 promoter -SBE2-KOD** |  |  |
| Forward | 5’- TTGGCCTGCGTCCTTAGTTTGCTGT -3’ | IGE GuangZhou |
| Reverse | 5’- GATGGGTAATCTTTCCTGGCAGGGG -3’ | IGE GuangZhou |
| **H4K5-KOD** |  |  |
| Forward | 5’- GGCGGAAAAGGCTTAGGCAAAGGGG -3’ | IGE GuangZhou |
| Reverse | 5’- TCCTCTGCCGGACATGGCATAATCT-3’ | IGE GuangZhou |
| **Supplemental Table S2. Antibodies used in this study.**   \| **PEAGENT or RESOURCE** \| **SOURCE** \| **IDENTIFIER** \| \| --- \| --- \| --- \| \| **Antibodies** \|  \|  \| \| CSRP2BP \| LSbio \| Catalogue #: LS-C349099-200 \| \| N-cadherin \| Cell Signaling Technology \| Catalogue #: 14215 \| \| N-cadherin(D4R1H)XP rabbit mAb \| Cell Signaling Technology \| Catalogue #: 13116S \| \| HPV16 E6/18 E6(C1P5） \| Santa cruz bio \| Catalogue #: sc-460 \| \| Anti- HPV 18 E7 \| Abcam \| Catalogue #:ab100953 \| \| Ki-67 Antibody \| Santa cruz bio \| Catalogue #: sc**-**15402 \| \| E-cadherin(4A2) Mouse mAb \| Cell Signaling Technology \| Catalogue #: 14472 \| \| Anti-Histone H4 \| Abcam \| Catalogue #: ab9051 \| \| Histone H4ac (pan-acetyl) antibody (pAb) \| Active Motif \| Catalogue #: 39925 \| \| anti-histone H4 (acetyl k16) antibody \| Abcam \| Catalogue #: ab109463 \| \| anti-histone H4(acetyl K12) antibody \| Abcam \| Catalogue #: ab46983 \| \| anti-histone H4 (acetyl K8) antibody \| Abcam \| Catalogue #: ab15823 \| \| anti-histone H4 (acetyl K5) antibody \| Abcam \| Catalogue #: ab51997 \| \| Histone H3 Rabbit mAb \| Cell Signaling Technology \| Catalogue #: 4499s \| \| Anti-acetyl-Histone H3 Antibody \| Merck Millipore \| Catalogue #: 06-599 \| \| anti-MMP2 antibody \| Abcam \| Catalogue #: ab97779 \| \| Anti-MMP-9 \| Abcam \| Catalogue #: ab38898 \| \| AKT \| Cell Signaling Technology \| Catalogue #: 4691 \| \| phospho-AKT \| Cell Signaling Technology \| Catalogue #: 4060 \| \| PI3 Kinase p85 Rabbit mAb \| Cell Signaling Technology \| Catalogue #: 4257T \| \| P-PI3K Rabbit Ab \| Cell Signaling Technology \| Catalogue #: 4228T \| \| Smad4(B-8) \| Santa cruz bio \| Catalogue #: sc-7966 \| \| Goat pAb to IgG (Alexa Fluor® reg 488) \| Abcam \| Catalogue #: ab150117 \| \| Goat Anti-Rabbit IgG H&L (Alexa Fluor® 594) \| Abcam \| Catalogue #: ab150080 \| \| Anti-HA tag antibody \| Abcam \| Catalogue #: ab18181 \| \| Flag-antibody \| Abcam \| Catalogue #: ab18230 \| \| Human IgG Antibody \| Proteintech \| Catalogue #: 10284-1-AP \| \| p16INK4a \| Abcam \| Catalogue #: ab16123 \| \| CD44 Antibody  Rabbit Polyclonal \| Proteintech \| Catalogue #: 15675-1-AP \| \| anti-GAPDH antibody[6C5] loading control \| Abcam \| Catalogue #: ab8245 \|   **Supplemental Table S3. Primers for Real-time PCR**   \| **Oligonucleotides** \| **Sequence** \| **Companies** \| \| --- \| --- \| --- \| \| GAPDH-F: \| 5’- GCATCCTGGGCTACACTGAG -3’ \| IGE GuangZhou \| \| GAPDH-R: \| 5’- CCACCACCCTGTTGCTGTAG -3’ \| IGE GuangZhou \| \| CSRP2BP-F: \| 5’-ACAAGCCCATCTCCTTCTCC -3’ \| IGE GuangZhou \| \| CSRP2BP-R: \| 5’-ACTCCATCGCCTTCCATCTC -3’ \| IGE GuangZhou \| \| N-cadherin-F: \| 5’-GCCCAAGACAAAGAGACCCA -3’ \| IGE GuangZhou \| \| N-cadherin-R: \| 5’-ACCCAGTCTCTCTTCTGCCT -3’ \| IGE GuangZhou \| \| E-cadherin-F: \| 5’- GGTGCTCTTCCAGGAACCTC -3’ \| IGE GuangZhou \| \| E-cadherin-R: \| 5’- GAAACTCTCTCGGTCCAGCC -3’ \| IGE GuangZhou \| \| MMP9-F: \| 5’-TTGACAGCGACAAGAAGAAGTGG-3’ \| IGE GuangZhou \| \| MMP9-R: \| 5’-GCCATTCACGTCGTCCTTAT -3’ \| IGE GuangZhou \| \| MMP2-F: \| 5’-ATGACAGCTGCACCACTGAG -3’ \| IGE GuangZhou \| \| MMP2-R: \| 5’-ATTTCTTGCCCAGGAAAGTG -3’ \| IGE GuangZhou \| | | |

**Supplemental Table S4. Primer for ChIP-PCR.**

| **Oligonucleotides** | **Sequence** | **Companies** |
| --- | --- | --- |
| E-box-F: | 5’- GAGCTGAAGGTGCGAGCTCC - 3’ | IGE GuangZhou |
| E-box-R: | 5’- ACTAAGGACGCAGGCCAAGC - 3’ | IGE GuangZhou |
| SBE1-F: | 5’- CACAGCAGCCGCCTCCACAC -3’ | IGE GuangZhou |
| SBE1-R: | 5’- CGGGACTCGCACCAGGAGTA -3’ | IGE GuangZhou |
| SBE2-F: | 5’- GCTAGGATGAGCTTGGCCTG -3’ | IGE GuangZhou |
| SBE2-R: | 5’- GGGAGCCTAACGTACGCTTC -3’ | IGE GuangZhou |

**Supplemental Table S5. Correlation between CSRP2BP expression and clinicopathologic features of cervical cancer (N=208).**

| **Characteristics** | **N** | **Percentage (%)** | **CSRP2BP expression** | | | | | ***P Value*** |
| --- | --- | --- | --- | --- | --- | --- | --- | --- |
|  |  |  | **High (n=107)** | | **Low or no (n=101)** | | |  |
| **Age** |  |  |  | |  | | | P=0.095 |
| <45 | 110 | 52.9 | 51 | | 59 | | |  |
| ≥45 | 98 | 47.1 | 56 | | 42 | | |  |
| **FIGO stage** |  |  |  | |  | | | *P<0.001* |
| I | 138 | 66.3 | 59 | | 79 | | |  |
| II | 70 | 33.7 | 48 | | 22 | | |  |
| **HPV** |  |  |  | |  | | | *P<0.001* |
| 16+18 | 141 | 67.8 | 98 | | 43 | | |  |
| Others ^a^ | 67 | 32.2 | 9 | | 58 | | |  |
| **Types of tumor growth** |  |  |  | |  | | | *P<0.001* |
| Exophytic | 107 | 51.4 | 36 | | 71 | | |  |
| Ulcerative | 80 | 38.5 | 50 | | 30 | | |  |
| Endophytic | 21 | 10.1 | 21 | | 0 | | |  |
| **Tumor size** |  |  |  | |  | | | *P<0.001* |
| <4 | 124 | 59.6 | 47 | | 77 | | |  |
| ≥4 | 84 | 40.4 | 60 | | 24 | | |  |
| **SCC level** |  |  |  | |  | | | P=0.069 |
| <1.5 | 118 | 56.7 | 54 | | 64 | | |  |
| ≥1.5 | 90 | 43.3 | 53 | | 37 | | |  |
| **Differentiation grade** |  |  |  | |  | | | P=0.309 |
| G1 | 10 | 4.8 | 3 | | | 7 | |  |
| G2 | 72 | 34.6 | 40 | 32 | | |  | |
| G3 | 126 | 60.6 | 64 | 62 | | |  | |
| **Stromal invasion** |  |  |  |  | | | *P<0.001* | |
| <1/2 | 99 | 47.6 | 33 | 66 | | |  | |
| ≥1/2 | 109 | 52.4 | 74 | 35 | | |  | |
| **Lymphovascular space invasion** |  |  |  |  | | | *P=0.026* | |
| No | 180 | 86.5 | 87 | 93 | | |  | |
| Yes | 28 | 13.5 | 20 | 8 | | |  | |
| **Pelvic lymph node metastasis** |  |  |  |  | | | P=0.653 | |
| No | 145 | 69.7 | 73 | 72 | | |  | |
| Yes | 63 | 30.3 | 34 | 29 | | |  | |
| **Recurrence** |  |  |  |  | | | P=0.714 | |
| No | 172 | 82.7 | 87 | 85 | | |  | |
| Yes | 36 | 17.3 | 20 | 16 | | |  | |
| **Vital status at follow-up** |  |  |  |  | | | *P=0.001* | |
| Death | 29 | 13.9 | 23 | 6 | | |  | |
| Alive | 179 | 86.1 | 84 | 95 | | |  | |

**^a^** HPV 58 (+) 24 cases, HPV 52 (+) 28 cases, HPV 33 (+) 10 cases, HPV 31 (+) 5 cases.

*P<0.05* was considered statistically significant.

**Supplemental Table S6. Multivariate analysis of prognostic factors for cervical cancer patients.**

| Outcomes | Variable | Exp(B) | *P* | 95%CI |
| --- | --- | --- | --- | --- |
| PFS | Tumor size  Stromal invasion  LVSI  CSRP2BP expression  FIGO stage | 1.455  1.571  3.545  2.655  3.170 | 0.347  0.342  ***0.019***  ***0.046***  ***0.005*** | 0.666-3.175  0.618-3.993  1.230-10.223  1.017-6.930  1.405-7.153 |
|  | Variable | Exp(B) | *P* | 95%CI |
| OS | Tumor size  Stromal invasion  LVSI  CSRP2BP expression  FIGO stage | 1.498  1.383  3.851  2.690  3.403 | 0.313  0.499  ***0.013***  ***0.044***  ***0.003*** | 0.684-3.281  0.540-3.543  1.328-11.170  1.029-7.034  1.499-7.726 |

Abbreviations: LVSI=lymphovascular space invasion; CI=confident interval; OS=overall survival; PFS=progression-free survival. *P<0.05* was considered statistically significant.

**Supplemental Table S7. Laboratory apparatus used in this study**

| **Name** | **Company and Model** |
| --- | --- |
| Laser confocal microscope | OLYMPUS: FV1000 |
| Landing centrifuge | Japan's Hitachi:LR21N |
| Microporous plate luminous detector | Germany BERTHOLD:LB942 |
| Fluorescence quantitative PCR instrument | Germany Agilent:MX3000P |
| PCR instrument | Germany Agilent: sure cycles8800 |
| Gel imager | Tanon Science & Technology Co., Ltd:Tanon-2500 |
| Uv-visible spectrophotometer | Japanese island ferry: UV-2550 |
| Carbon dioxide incubator | US Thermo: Forma3131 |
| Cryogenic refrigerator | Haier: DW-86L386 |
| Vertical electrophoresis tank | Tanon Science & Technology Co.,Ltd:VE-180 |
| Electrophoresis apparatus | Tanon Science & Technology Co.,Ltd: EPS-600+HE-120+VE |
| Inverted fluorescence study stage microscope | Japanese OLYMPUS:IX71+DP72+cellsense |
| Biosafety cabinet | SingaporeESCO:AC2-4S1 |
| Overspeed sorting flow cytometer | US BD: FACS AriaII |
