# Supplementary figures and images for: Histone acetyltransferase CSRP2BP promotes the epithelial–mesenchymal transition and metastasis of cervical cancer cells by activating N-cadherin
Source: J Exp Clin Cancer Res. 2023 Oct 17;42:268. doi: 10.1186/s13046-023-02839-2 (PMC10580587; doi:10.1186/s13046-023-02839-2)

**high**

**low**

**no/untected**

**T**

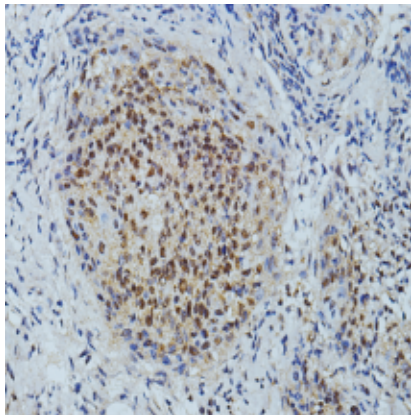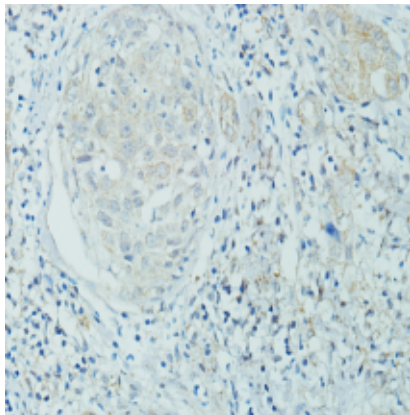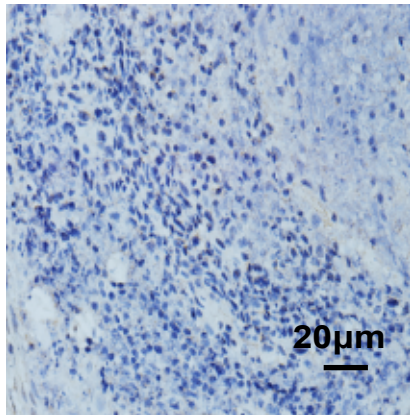

**20μm**

Supplement: Supplementary file 2 — Additional file 2: Supplemental Figure S1. Expression of CSRP2BP in cervical cancer. Representative images of IHC staining for CSRP2BP in paraffin embedded sections of cervical cancer patients. [file 13046_2023_2839_MOESM2_ESM.pdf]

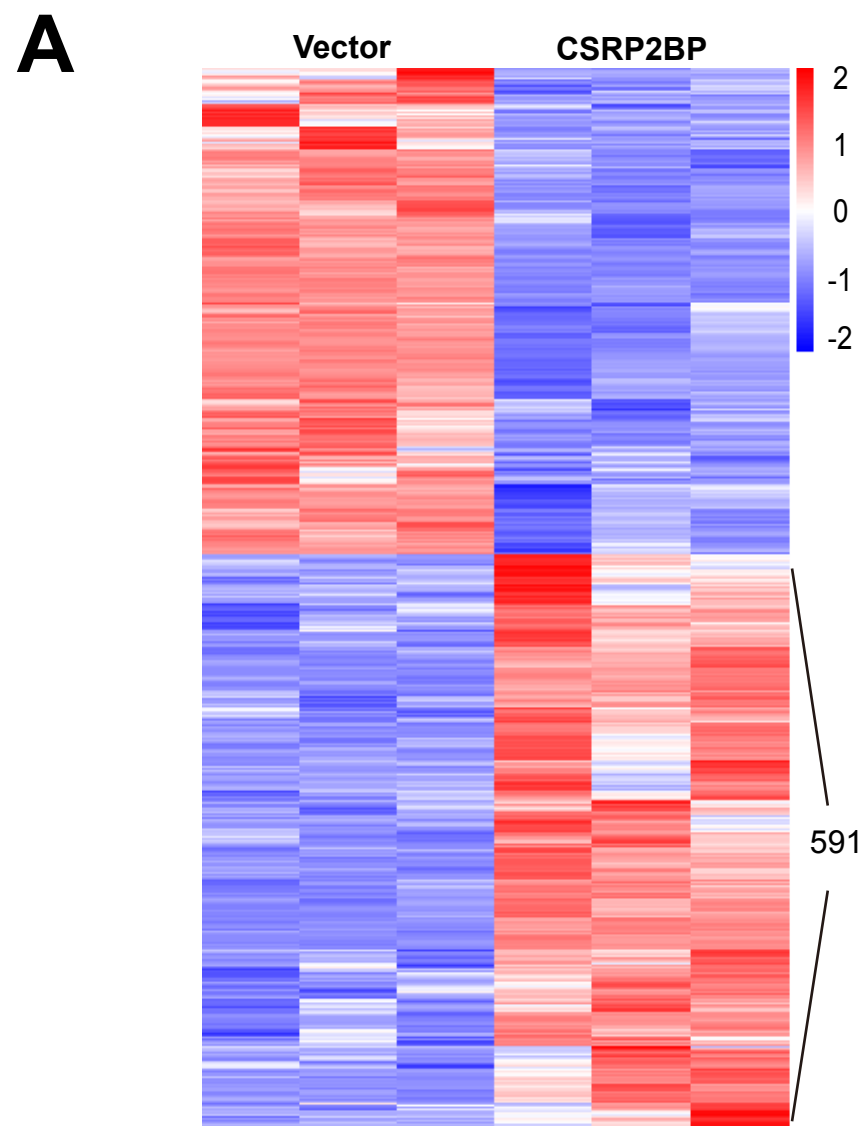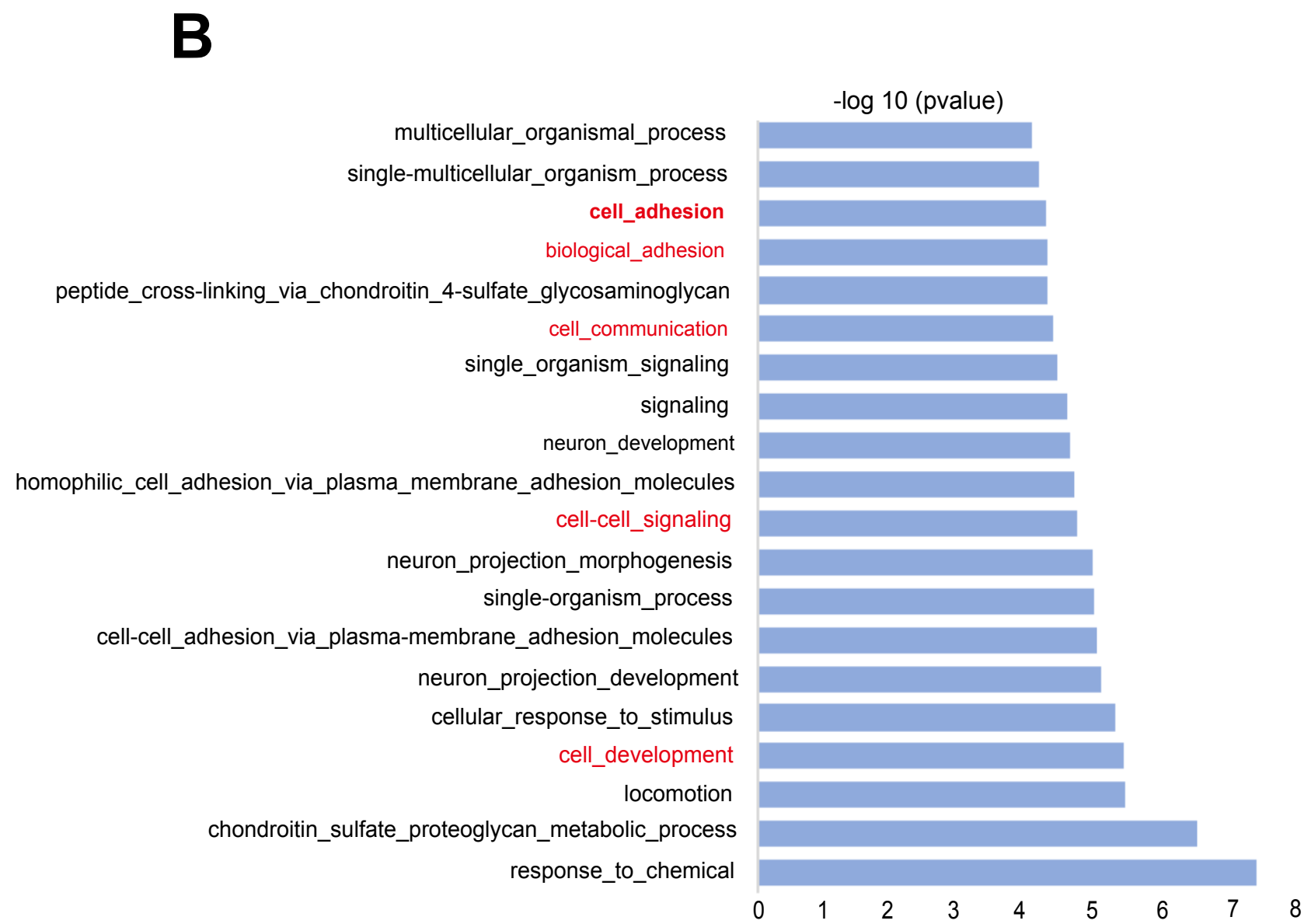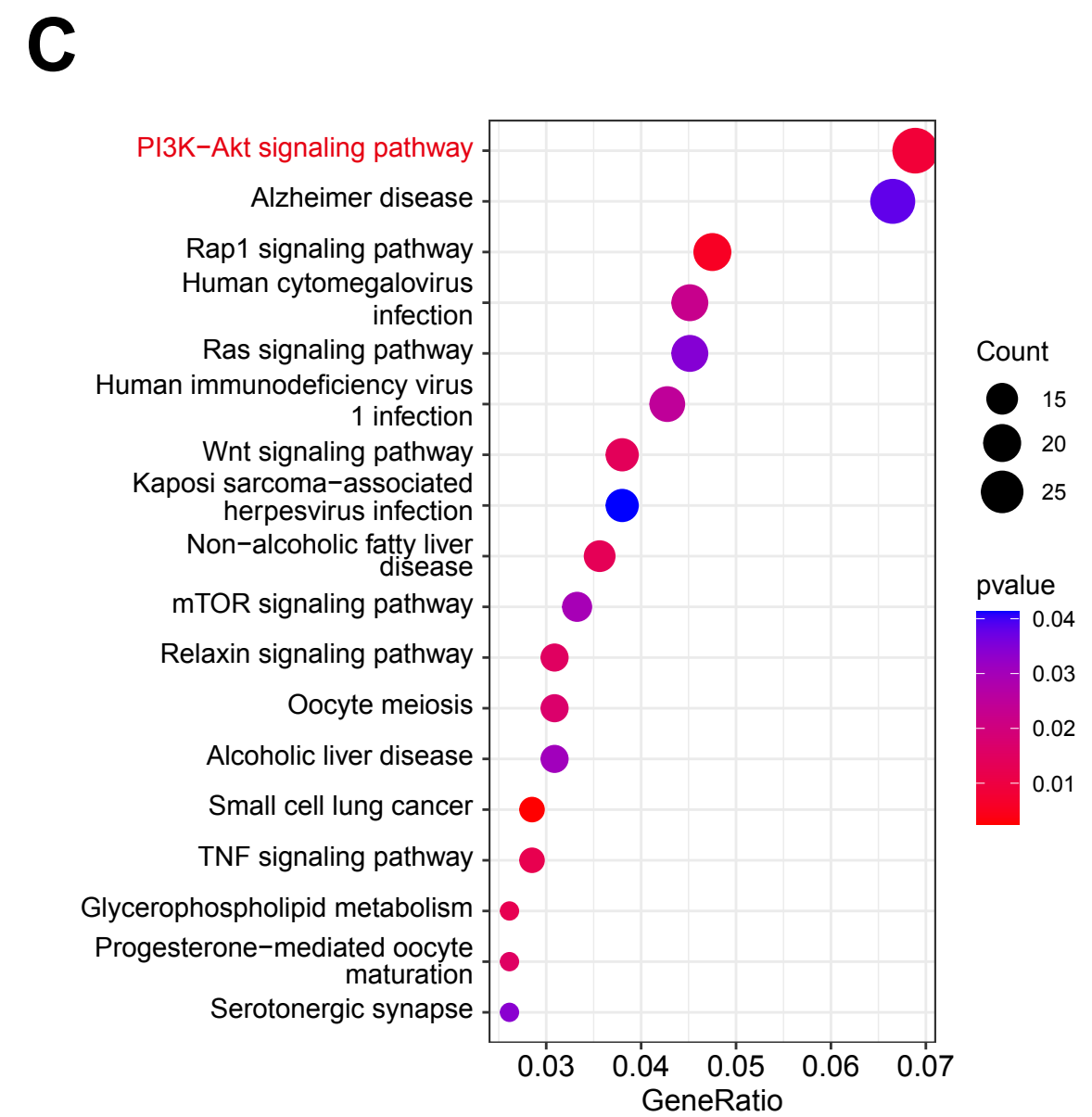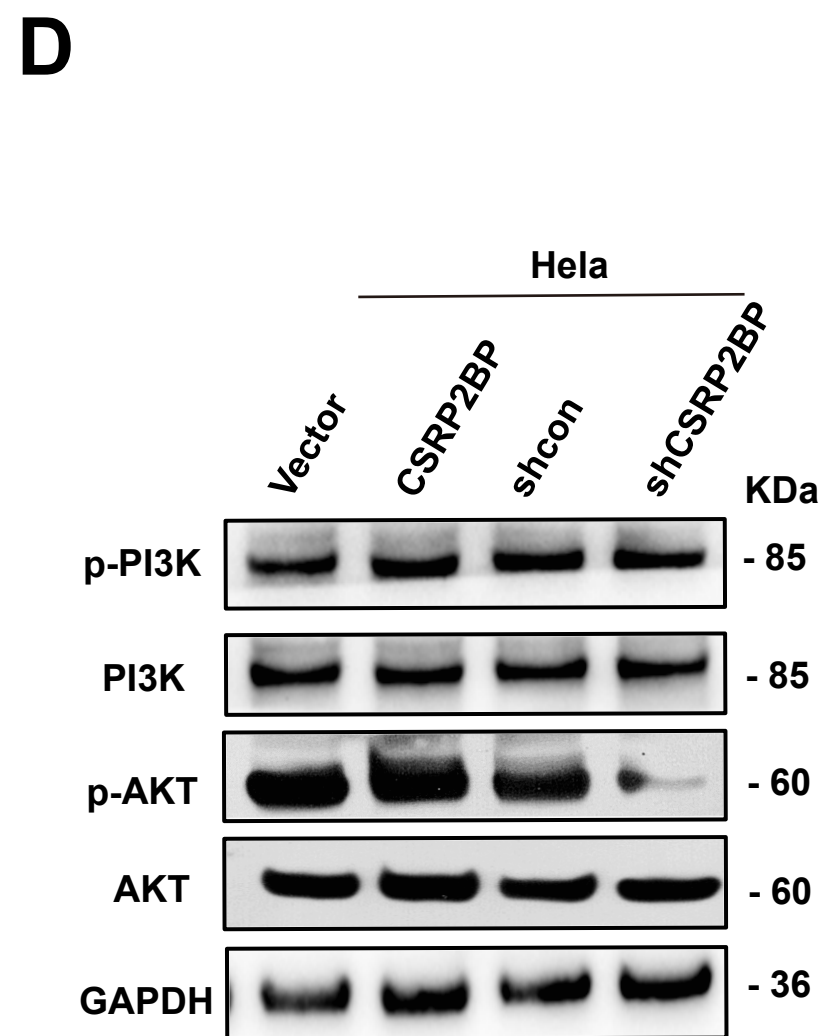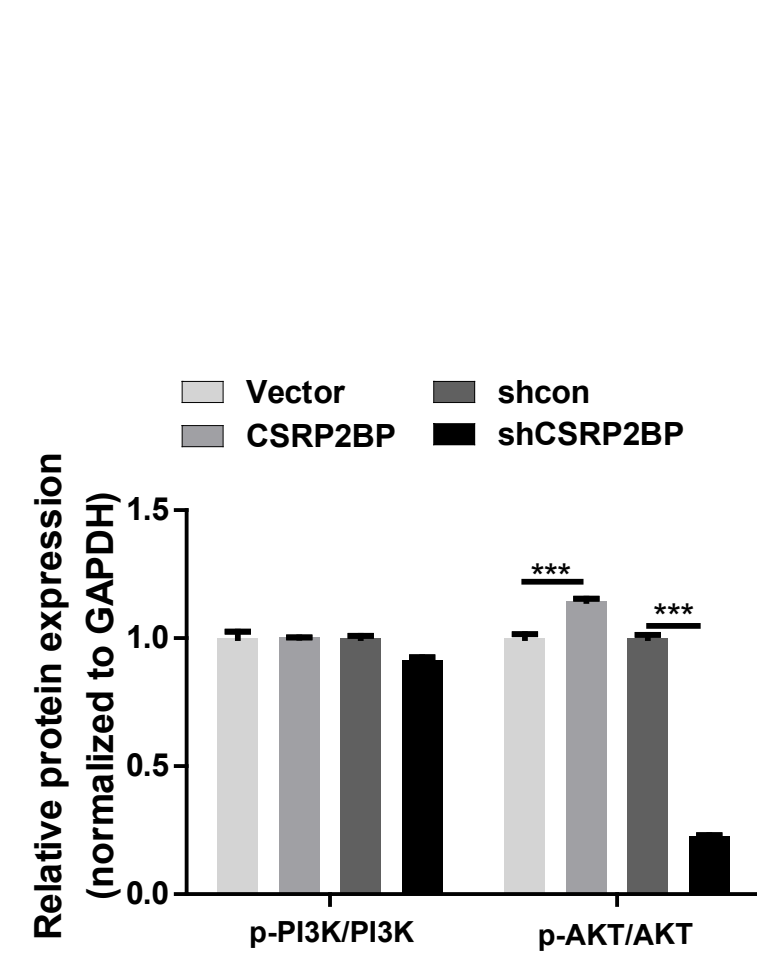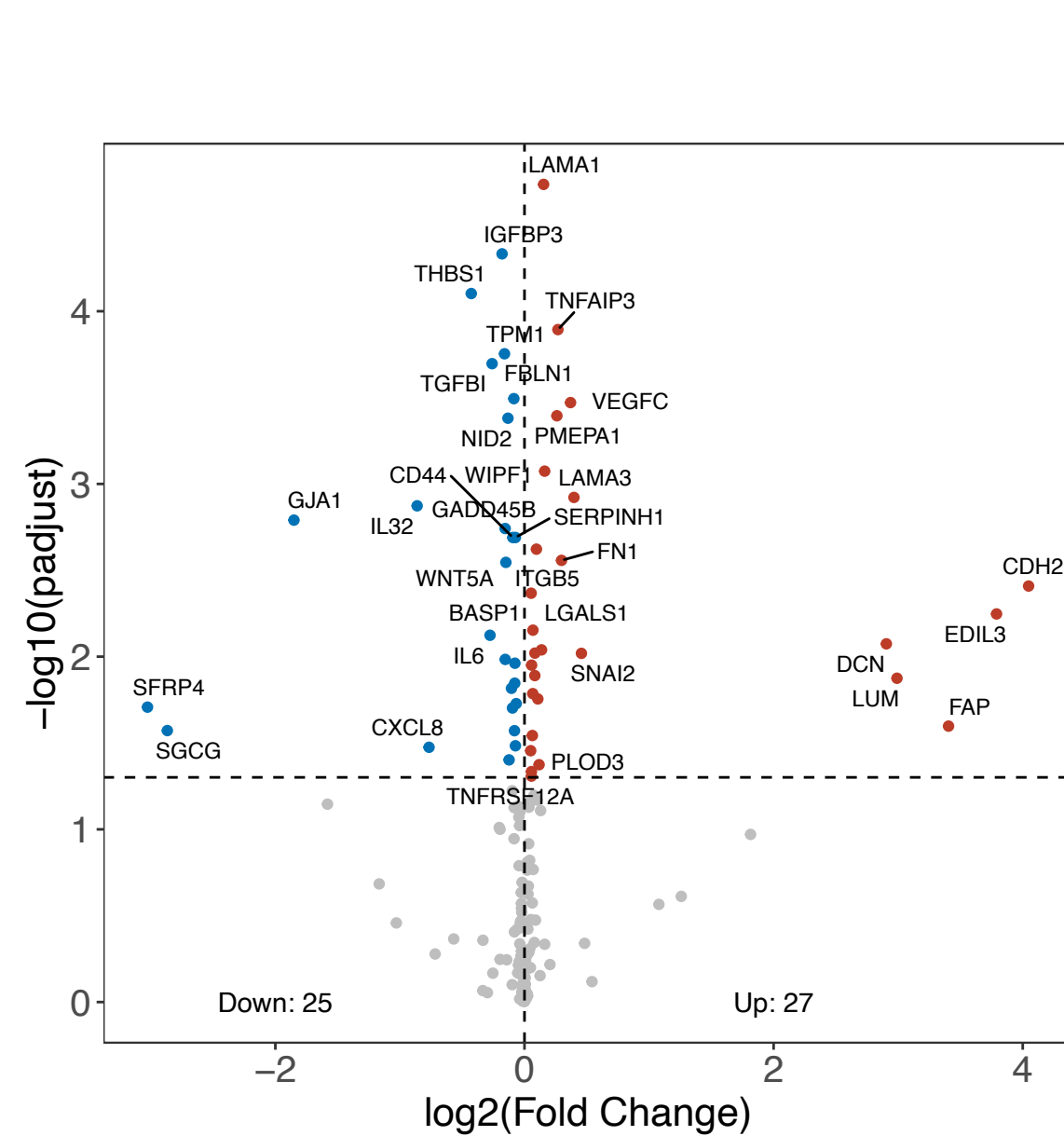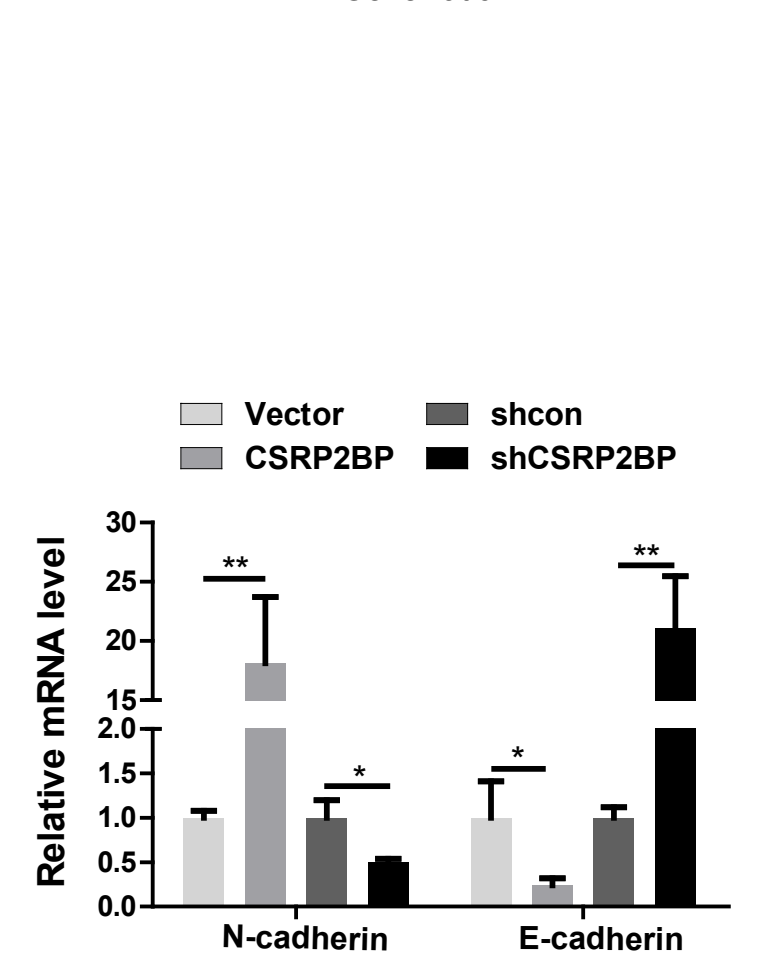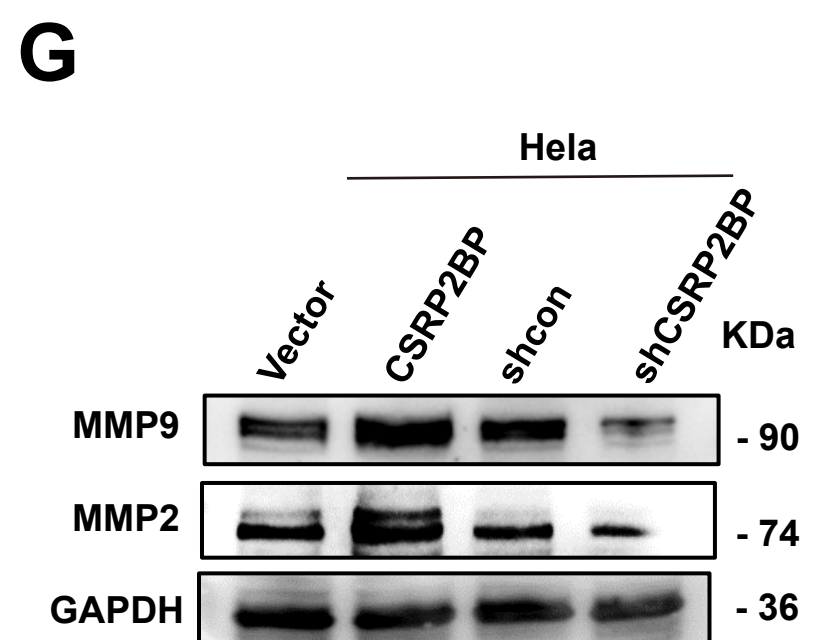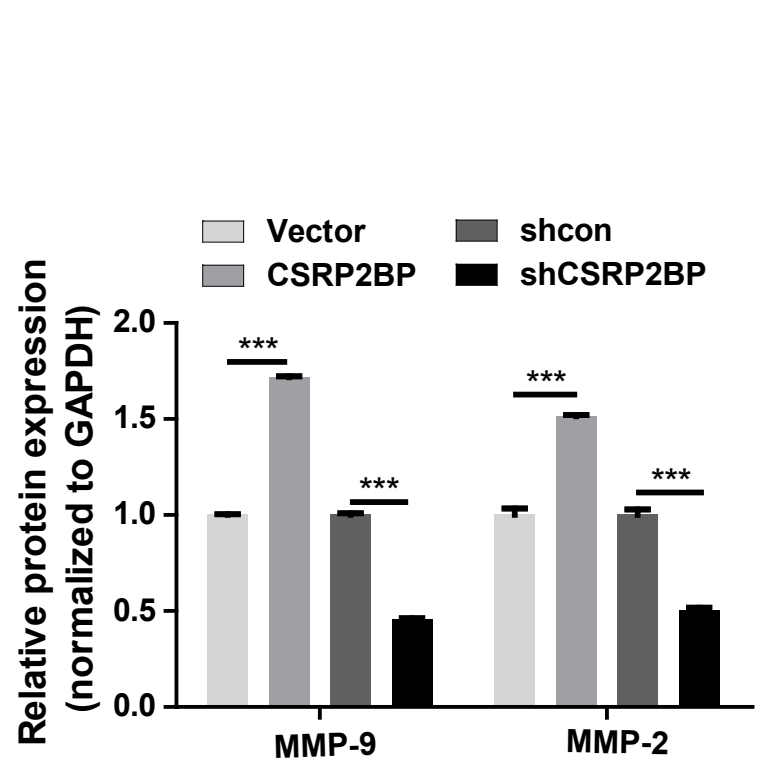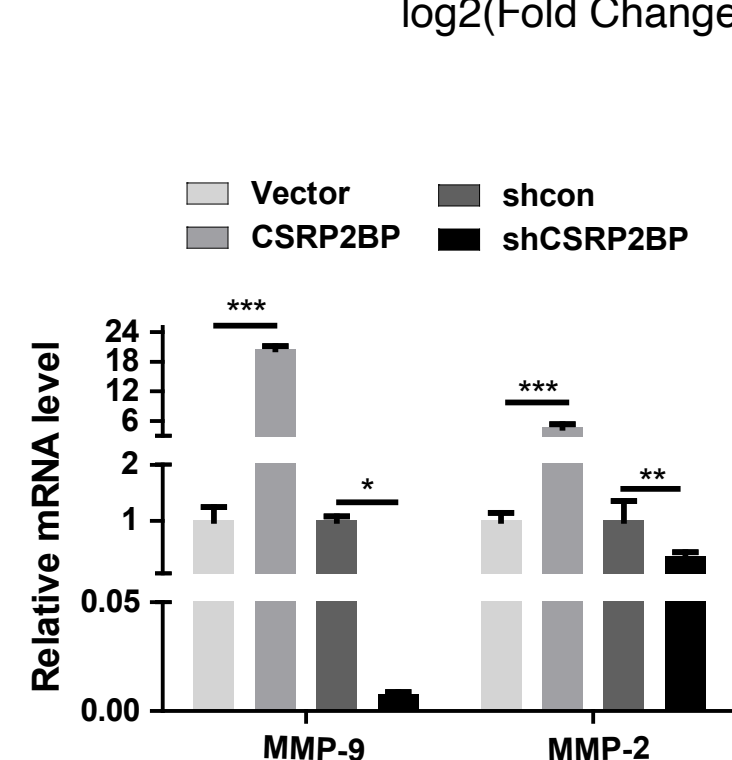

Supplement: Supplementary file 3 — Additional file 3: Supplemental Figure S2. CSRP2BP induces EMT-related signals in Hela cells. (A) Heatmap representation of up-regulated and down-regulated genes in Hela-CSRP2BP cells compare with control group. (B) GO analysis of top 20 biological functions of up-regulated genes in Hela-CSRP2BP cells. (C) KEGG pathways enriched by the selected up-regulated genes. (D) The protein expression levels of the PI3K/AKT signalling pathway were detected in Hela-CSRP2BP cells, Hela-shCSRP2BP and the respective control cells by Western blotting. (E) Volcano Plots of up-regulated and down-regulated genes in Hela-CSRP2BP cells compare with control group. (F) The mRNA expression of N-cadherin and E-cadherin in Hela-CSRP2BP and Hela-shCSRP2BP cells were measured by RT-PCR. Statistical analysis was shown as mean SD (n = 3). (G) The protein expression levels of EMT-related markers were measured by Western blotting. (H) The mRNA expression levels of EMT-related markers were detected by RT-PCR. Statistical analysis is shown as mean ± SD (n = 3). (*P < 0.05, **P < 0.01, ***P < 0.001). [file 13046_2023_2839_MOESM3_ESM.pdf]

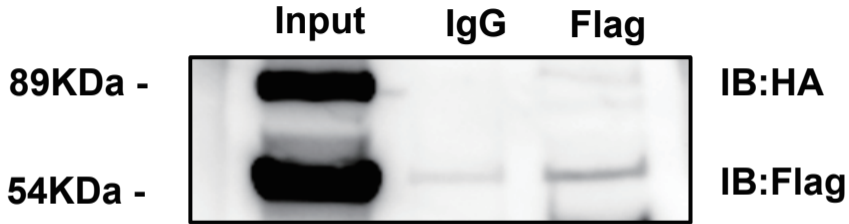

**Input:Flag-HA-smad3+HA-CSRFP2BP**

Supplement: Supplementary file 4 — Additional file 4: Supplemental Figure S3. CSRP2BP does not bind to SMAD3 in Hela cells. The interaction between HA-tagged CSRP2BP and HA & flag-tagged SMAD3 was tested by Co-IP assay with anti-flag antibody or control normal IgG. Western blotting was stained with anti-HA antibody. [file 13046_2023_2839_MOESM4_ESM.pdf]
